# The Neuro-Melanoma Singularity: Convergent Evolution of Neural and Melanocytic Networks in Brain Metastatic Adaptation

**DOI:** 10.3390/biom15121683

**Published:** 2025-12-02

**Authors:** Vlad-Petre Atanasescu, Alexandru Breazu, Stefan Oprea, Andrei-Ludovic Porosnicu, Anamaria Oproiu, Mugurel-Petrinel Radoi, Octavian Munteanu, Cosmin Pantu

**Affiliations:** 1 Faculty of General Medicine, “Carol Davila” University of Medicine and Pharmacy, 050474 Bucharest, Romania; vladpetre.atanasescu@drd.umfcd.ro (V.-P.A.); andreiiporosnicu@umfcd.ro (A.-L.P.); cosmin.pantu@umfcd.ro (C.P.); 2 University Hospital, Faculty of Medicine (Faculty of General Medicine), “Carol Davila” University of Medicine and Pharmacy, 010024 Bucharest, Romania; 3 Puls Med Association, 051885 Bucharest, Romania; 4 Department of Anatomy, “Carol Davila” University of Medicine and Pharmacy, 050474 Bucharest, Romania; 5 Elias Emergency University Hospital, Faculty of Medicine (Faculty of General Medicine), Carol Davila University of Medicine and Pharmacy, 020021 Bucharest, Romania; 6 Department of Neurosurgery, “Carol Davila” University of Medicine and Pharmacy, 050474 Bucharest, Romania; 7 Department of Vascular Neurosurgery, National Institute of Neurology and Neurovascular Diseases, 077160 Bucharest, Romania

**Keywords:** melanoma brain metastasis, neural crest reactivation, synaptic mimicry, gliovascular coupling, electro-metabolic integration, mitochondrial networks, adaptive coherence, precision dissonance

## Abstract

Melanoma cells in the brain may use similar mechanisms for adapting to injury and/or disease (that is, through continued reallocation of energy, matter, and information) as other cell types do to create an environment in which cancer cells can grow and sustain themselves within the confines of the brain. These adaptable mechanisms include the ability to reactivate dormant neural crest-derived migration and communication pathways. Unlike some other types of cancers that invade neural tissue as a simple invasion, melanomas are capable of achieving limited molecular, metabolic, and electrical similarity to the neural circuitry of the brain. Melanomas achieve this limited similarity through both vascular co-optation and mimicking synaptic functions, as well as through their engagement of redox-coupled metabolic pathways and feedback-regulated signal transduction pathways. The result is the creation of a metastable tumor–host system, where the relationship between tumor and host is defined by the interaction of stabilizing and destabilizing forces; forces that define the degree of coherence, vulnerability, and persistence of the tumor–host system. In this review, we integrate molecular, electrophysiological, and anatomical data to develop a single unifying hypothesis for the functional integration of melanoma cells into the neural tissue of the brain. Additionally, we describe how neural crest-based regulatory pathways are reactivated in the adult brain and how tumor–host coherence is developed as a function of the shared thermodynamic and informational constraints placed on both tumor and host. We also describe how our proposed conceptual model allows for the understanding of therapeutic interventions as selective disruptions of the neural, metabolic, and immunological couplings that support metastatic adaptation.

## 1. The Emergence of Neuro-Melanoma Convergence

Melanoma retains a “developmental memory” because it is derived from the neural crest. The neural crest is an embryonic population that develops into the peripheral nervous system (PNS), peripheral glia, and craniofacial mesenchyme. The neural crest’s developmental history provides melanoma cells with characteristics that favor movement, flexibility, and excitability, which are more pronounced when melanoma cells enter the central nervous system (CNS) [1]. The CNS is a unique environment that has high concentrations of various neurotransmitters, ions, and growth factors and has a specialized vascular basal lamina and multiple types of glia that are able to rapidly respond to and integrate different signals. In this environment, melanoma cells reactivate many of their original neuroectodermal developmental programs (axon guidance systems, vesicular release apparatus, etc.) that were present during their neural development [2]. These developmental programs support melanoma cell adaptation upon CNS entry rather than being indicative of a shift towards a hypothetical identity [3]. Upon entering the CNS, melanoma cells initially contact cerebrovascular endothelial cells through selectins and integrins. Selective contact occurs at areas with decreased shear forces and varying expression of tight junctions. Chemokines like CXCL12-CXCR4, CCL2-CCR2, and CX3CL1-CX3CR1 direct transendothelial migration and the local production of MMP2, MMP9, and heparanase break down the basement membrane [4]. Adhesion of ICAM1 and VCAM1 to melanoma cell integrins creates anchor points, and most intracranial melanomas use vascular co-optation rather than induce angiogenesis. Melanoma cells migrate along capillaries and arterioles and utilize pericytes and astrocytic endfeet as structural rails, with laminin isoform and β1-integrin signaling that couple extracellular stimuli to cytoskeletal regulators [5,6].

Once established within the CNS, melanoma cells interact with a neural environment designed for maintaining calcium homeostasis and redox balance. They have a wide range of neurotransmitter receptors and ion channels (e.g., AMPA/NMDA receptor subunits, mGluR, HCN, KCNQ, T-type/L-type calcium channels, STIM1/ORAI1, TRPM7/TRPV4) that allow them to control motility, proliferation, and metabolic activity through CaMKII-CREB, calcineurin-NFAT, and MAPK signaling cascades [7]. Maintaining chloride homeostasis (NKCC1/KCC2) allows melanoma cells to regulate their volume in confined spaces and modulate their membrane potentials via sodium-dependent actin remodeling through Rho GTPases [8]. Additional axon guidance cues (SLIT-ROBO and netrin-DCC/UNC5) help to define the direction of melanoma cell migration through the brain parenchyma and reflect conserved developmental logic that is used in a malignant context [9].

Interpretive scope and evidence status. The “neural-melanoma convergence” is at an early point in time to identify as a mechanistic spectrum as opposed to a full neural integration theory. A number of components of the convergence are supported by data from melanoma metastases into the brain; these include neural crest lineage reactivations in the brain, melanoma cells colonizing around blood vessels and co-opting them, the expression of receptors and ion channel programs used by neurotransmitters, and activity-dependent changes in intracellular Ca^2+^ and metabolism in organotypic or intracranial models [10]. These findings provide strong evidence that melanoma may develop partial biochemical, bioelectric, and metabolic compatibility with the CNS niches. However, the more general interpretations of melanoma acquiring stable bidirectional synaptic plasticity with neurons, or long-lasting circuit-level “integration”, should be considered working hypotheses until they have been tested by melanoma-specific ultrastructure studies, electrophysiology, and perturbations of causal mechanisms [11]. Therefore, throughout this review, we will use integrative terms (coupling, coherence, entrainment) as conceptual shorthand for the graded tumor–host interactions but will indicate where our interpretation extends beyond the current evidence on melanoma.

Melanoma cells form functional connections with neurons. Electron microscopy studies demonstrate that melanoma cells form junctions with postsynaptic density-like scaffold and synaptic vesicle protein on the tumor side, and partial synchronization of melanoma cells and adjacent neuronal processes is observed with calcium imaging [12]. Mechanisms underlying these interactions may be glutamatergic spillover sensed via AMPA/NMDA receptors, ephaptic coupling, connexin-mediated gap junctions (Cx43/Cx30), and activity-dependent release of neuroligin-like ligands. Additionally, cholinergic and adrenergic signaling pathways may modulate the melanoma cell cycle and motility through cAMP-PKA signaling [13]. These interactions facilitate low-amplitude, long-term entrainment of melanoma cells with local neural circuits. Astrocytes reinforce the microenvironment created by melanoma cells. Astrocytes enhance glutamate uptake via EAAT1/2 and provide a lactate shuttle to increase energy availability for nearby neurons and release exosomes with miR-19a/miR-21 and metabolic enzymes that inhibit PTEN and activate PI3K-AKT signaling under oxidative stress [14,15]. Microglia that are recruited to the site of melanoma through CSF1R and TREM2 and are exposed to C3 adopt a supportive phenotype characterized by IL-10 and TGF-β release, extracellular matrix remodeling, synaptic pruning, and reduction in cytotoxic signals [16]. Pericytes regulate capillary tone and send PDGFRβ-mediated signals to maintain a stable microenvironment with consistent delivery of oxygen and metabolites [17].

As a result of continued interaction between melanoma cells and their surrounding neural environment, spatially organized patterns emerge at the neuro-melanocytic interface. Single-cell and spatial maps demonstrate gradients of neuronal genes expressed in response to melanoma in radial directions away from the center of the tumor, and peritumor cells frequently exhibit melanoma-specific transcriptional programs [18]. It appears that melanoma cells transition between two distinct states: a proliferative/depolarized state associated with glutamatergic input and CaMKII-CREB activity and a migratory/mechanically responsive state defined by enhanced TRP and integrin signaling and dynamic chloride regulation [19]. Transitions between these states are influenced by neuronal activity, circadian rhythm, and local inflammation. Resistance to immune checkpoint inhibitors typically correlates with the expression of synaptic inhibitory molecules (neuroligin–neurexin pairs, GABA_A receptor subunits, neuropilin-2) that reduce the effectiveness of immune effector cells [20]. Additionally, the presence of specific ion channel profiles confers melanoma cells with metabolic and DNA repair resistance contributing to resistance to therapy, whereas radiotherapy-induced glutamate and ATP release, astrocyte activation, and increased ephaptic coupling may contribute to residual tumor coherence [21]. Understanding these interactions will lead to novel therapeutic strategies such as blocking AMPA/NMDA receptors, inhibiting MCT-mediated metabolite transfer, disrupting gap junctions, and modulating chloride transport [22].

Traditionally, the management of intracranial melanoma was based on vascular permeability and immune evasion. Current data suggest a more integrated process in which melanoma cells incorporate design principles from the neural network and participate in circuit-level interactions [23]. This perspective does not suggest intentionality but highlights the common developmental logic employed by melanoma cells and neurons, where melanoma utilizes properties developed for perception and plasticity in one context to enable metastasis in another. Viewing melanoma cells as participating in functional integration will establish a clearer link between molecular mechanisms and clinical behaviors [24].

### Purpose, Methods, and Organization of This Review

This review seeks to establish a mechanistic explanation of how melanoma brain metastases interact with neural circuits. The goal of this review is to compile recent molecular, electrophysiological, metabolic, and spatial data into a unified model that describes intracranial adaptations and identifies opportunities for therapeutic intervention. Rather than focusing on catalogs of pathways, the focus will be on mechanisms of communication (ionic, metabolic, and vesicular).

Recent studies using high-resolution or causally informative methodologies (single-cell and spatial multi-omics, quantitative proteomics and metabolomics, live-cell and in vivo calcium imaging, electrophysiology, organotypic co-culture, and intracranial models that evaluate neuro-tumor interaction) are the basis for the synthesis presented in this review. Only studies that directly describe brain-specific adaptation or communication were considered.

Section 2 discusses the developmental origins of this phenomenon; it describes how the neural crest memory, chromatin accessibility, and some important transcriptional circuits (e.g., SOX10–MITF–BRN2 axis) predispose melanoma cells to re-express their neural programs when they migrate to the brain. Section 3 describes the brain as a selective environment and details the molecular choreography of crossing of the blood–brain barrier (BBB), perivascular colonization, and white matter migration, in addition to the role of endothelial cells and pericytes in stabilizing the melanoma cells’ phenotype. Section 4 considers the phenomenon of the melanoma cells mimicking synapses and integrating with electrical activity and details the various types of evidence for their ability to express synaptic receptors, ion channels, gap junctions, ephaptic communications, and transition between oscillatory states based upon changes in intracellular calcium levels and membrane potentials.

Section 5 investigates the dialog between glia and the immune system of the host, including how astrocyte-derived exosomes signal to other cells, how melanoma cells acquire glutamine and lactate, how microglia shift their phenotypes, and how C1q-dependent degradation remodels the immunogenicity and metabolism of the metastatic niche. Section 6 connects all of these threads through the use of multi-omic and spatially resolved data to present a preliminary “connectome” of metastasis; it outlines a map of the flow of information between melanoma cells, neurons, glia, and the vasculature. Section 7 applies the results of the previous sections to propose the principles of neuromodulatory oncology; it defines strategies that can disrupt the coherence of networks by modulating receptors and ion channels, by disrupting metabolic coupling, by using time-specific combinations of radiotherapy and immunotherapy, and by synchronizing the chronobiological rhythms of the host and the metastatic cells.

Finally, Section 8 is the concluding section, which reviews the major points discussed in the preceding sections, identifies areas of uncertainty in our current understanding, and proposes specific experimental approaches that can be used to test and refine this integrated model.

## 2. Developmental Roots: Neural Crest Memory and Lineage Reactivation

Melanoma’s capacity to demonstrate neural-like behaviors in the CNS stems not solely from mutations developed during tumor evolution or environmental selective pressures; it also results from the reactivation of conserved developmental programs that were encoded by the neural crest lineage of the tumor [25]. The neural crest is a transient embryonic progenitor cell population that gives rise to peripheral neurons, Schwann cells, melanocytes, and craniofacial mesenchymes. Their descendants have the capacity to migrate, integrate signals, and be electrophysiologically adaptive. Melanoma cells in the CNS engage the pre-existing lineage programs of their neural crest ancestors using genetic and epigenetic mechanisms, rather than developing entirely novel identities [26].

### 2.1. Legacy of Neural Crest Developmental Innovation

Cells produced by the neural crest migrate extensively, give rise to multiple cell types, and undergo an EMT-like transition mediated by morphogen gradients (WNT, BMP, FGF, NOTCH) and transcriptional regulators (SOX10, PAX3, FOXD3, MITF) [27]. As the cells migrate through the embryo, they interpret positionally dependent information through receptor tyrosine kinases (RTKs) (ERBB, FGFR, RET) and calcium-dependent adhesion systems [28]. Though adult melanocytes generally down-regulate many of the genes associated with the neural crest, their chromatin retains accessibility at neural crest-specific enhancer motifs, retaining H3K27ac peaks and SOX10/BRN2/MITF-responsive enhancers that maintain the embryonic regulatory capacity of the cells [29,30]. Rapidly upon being exposed to environmental stresses, such as hypoxia, inflammation, or morphogenic proteins similar to those present during neural crest migration, this chromatin organization is activated again. When melanoma cells migrate into the brain, they are therefore able to rapidly reactivate lineage intrinsic programs due to encountering morphogens and ECM structures similar to those encountered by migrating neural crest cells [31].

Additionally, neural crest-derived cells inherently possess electrophysiological properties through T-type Ca^2+^ channels, HCN channels, and TRPM7 conductances that produce rhythmic ionic oscillations controlling migration and differentiation [32]. Therefore, the presence of these channels in melanoma metastases indicates that the electrophysiological competency of melanoma cells is a lineage-inherited trait rather than a mutated trait that is newly acquired. Therefore, when melanoma cells enter the brain, they already possess a pre-configured regulatory system that will facilitate their neural adaptation [33].

While the lineage-primed chromatin landscape has been proposed as a possible substrate for the neural program re-expression in the brain; and while it is also indicated by melanoma brain metastasis data that there are many types of inter-lesional and intra-lesional heterogeneities within this dataset; and that melanoma BM may express varying degrees of neural crest lineage transcriptional re-opening as well as varying degrees of SOX10-MITF-BRN2 axis reorganization compared to both melanomas at extracranial sites and selected models of melanoma BM, the metastases in melanoma BM appear to be located along a spectrum of developmental states based on their clonal histories, their microenvironmental contexts, their treatments and local niche signals, so that some melanoma BM will have a high level of MITF expression indicating proliferation programs, others will have a high level of BRN2 expression indicating invasion programs, and others will express partial or unstable levels of either of these two axes [34,35]. Therefore, we view the neural crest memory as a potential facilitator as opposed to an absolute requirement, and further, we emphasize that the extent, direction, and temporal stability of lineage reactivation in melanoma BM is highly variable and requires continued melanoma-specific validation [36].

### 2.2. Transcriptional and Epigenetic Reactivation in the Brain Microenvironment

In the CNS, melanoma cells reactivate neural crest transcriptional states instead of dedifferentiating randomly. RNA-seq and ATAC-seq analyses demonstrate that melanoma cells reactivate enhancer sets that control axon guidance (SLIT2, ROBO1, SEMA3A), neurotransmission (GRIN2A, SYT1, SLC17A7), and synaptic structure (DLG4, NRXN1, NLGN3) [37,38]. Cycling between transcriptional states dominated by SOX10, MITF, and BRN2 produces a dynamic regulatory triad that controls cellular plasticity. Intracranially metastasized melanomas often establish a BRN2-high program that activates proneural factors (ASCL1, NEUROD1, DLX2), producing a hybrid state that combines motility, excitability, and metabolic versatility while maintaining malignancy [39].

Epigenetic remodeling reinforces the transcriptional changes described above. Neural-related enhancers are reactivated through histone acetylation and recruitment of neuron-specific SWI/SNF complexes (BAF53B and ACTL6B), allowing for enhancer–promoter looping. Methylation patterns of DNA change to demethylate genes involved in neurotransmitter release and neurite growth, and to hyper-methylate melanocytic differentiation loci (TYR, DCT, MLANA) [40]. The incorporation of histone H3.3 at dynamic promoters supports rapid transcriptional switching, leading to a metastable chromatin environment capable of oscillating between melanocytic and neuroadaptive programs. The post-transcriptional regulation of these states further stabilizes them: miR-182/183/96 regulate FOXO-driven redox responses, miR-211 regulates mitochondrial metabolism and Ca^2+^ signaling, and lncRNAs (BANCR, NEAT1, SAMMSON) act as scaffolds for BRN2-MITF interactions and coordinate enhancer activity [41,42]. Together, all of these mechanisms enable the reversible switching between the states necessary for neural mimicry [43].

Protein data confirm the existence of these findings. Intracranial metastases express synaptic proteins that are not commonly expressed in extracranial metastases (NLGN3, CNTNAP2, SYN1, STXBP1, VAMP2, RIM1), thereby allowing for the creation of pseudo-synaptic structures and the communication of tumors through neurotransmitters. The reactivation of lineage therefore enables metastasis by utilizing, rather than merely mimicking, neuronal machinery [44].

### 2.3. Environmental Stimulation and Functional Consequences of Lineage Reactivation

Neurons, astrocytes, and microglia stimulate the reactivation of neural crest lineage through morphogens and trophic factors, such as neuregulin-1, WNT7A, sonic hedgehog, BDNF, and GDNF, which activate ERBB3/ERBB2, AKT-CREB, MAPK-AP1, and integrin-mediated FAK-SRC-RAC1 signaling pathways [45,46,47]. These signaling pathways re-establish cellular polarity, regulate vesicle trafficking, and support perivascular migration patterns that are similar to the migration patterns observed during neural crest development. Brain extracellular ion concentrations (Ca^2+^, K^+^, glutamate) also drive patterned electrical activity in melanoma cells via ORAI1-STIM1 store-operated entry and TRPM7/TRPV4 mechanosensitive channels [48]. The resulting Ca^2+^ oscillations are similar to the synchronized activity observed in developing neural circuits and create a bioelectric communication network that supports the coordinated adaptation of tumors [49]. Electron microscopy has shown that intracranial metastases have neurite-like extensions rich in GAP43, MARCKS, and DCX that are aligned along axons, thus permitting vesicle transport and Ca^2+^ propagation [12]. Furthermore, the secretion of glutamate, D-serine, and ATP influences neuronal excitability and glial responses, thereby generating a hybrid neuro-melanocytic microcircuitry [50].

Therefore, in total, these processes produce metastatic populations that function as partially integrated neural subsystems—responsive to stimuli, capable of communicating at the level of circuits, and metabolically linked to glial networks [51]. The neural crest lineage provides the regulatory template and the functional capability for the integration of metastatic populations. Therefore, the concept of metastasis is redefined not as the result of random dedifferentiation but as the lineage-based reinstatement of conserved developmental programs [52]. Table 1 summarizes the multi-layered gene expression, epigenetic, and electrophysiological mechanisms that allow melanoma brain metastases to function as lineage-remembered neural crest-derived cells.

## 3. The Brain as a Selective Ecosystem: Molecular Choreography of Infiltration and Adaptation

While the brain’s vascular and immune environments protect it from metastasis with a high degree of restriction, the metabolic and electrical characteristics of the brain provide a similar developmental environment for melanoma cells to grow within. Melanoma successfully develops a niche in the brain by developing compatible interfaces with the brain’s vascular and cellular environments [58]. As a result of melanoma’s ability to interpret and adjust to the brain’s rhythmic patterns, what initially began as a vascular obstruction evolves into a process of molecular domestication where melanoma cells and neural tissues form compatible interfaces at the endothelial, glial, and neuronal levels [59].

### 3.1. Transendothelial Migration and Vascular Co-Option

Melanoma cells enter the brain environment and begin adapting to it when they transition from a blood vessel to the CNS. At the BBB, melanoma cells are able to convert hemodynamic signals into chemotactic responses and are arrested at sites of discontinuity in the tight junctions of the endothelial cells, where VCAM1, ICAM1, and E-selectin are expressed [10,60]. The melanoma cells then bind to the endothelial cells using α4β1, αvβ3, and α6β4 integrins and initiate signaling through the FAK-SRC, PI3K-RAC1, and RHOA-ROCK pathways, which lead to the polarization of the cytoskeleton and cycling of focal adhesions. Chemokines such as CXCL12-CXCR4, CCL2-CCR2, and CX3CL1-CX3CR1 guide the lamellipodia of melanoma cells to intercellular junctions, where the cells can secrete MMP2, MMP9, and heparanase locally to transiently disrupt the endothelial basement membrane [61]. The endothelial cells also release VEGF-A and Angiopoietin-2 to relax pericyte coverage, allowing melanoma cells to move through the paracellular spaces. Unlike other cancers, melanoma typically performs vascular co-option, which is migrating along existing vasculature, providing both oxygen and architectural support [62].

Strength of relative evidence. The BBB-to-perivascular sequence of steps discussed above is supported by differing degrees of evidence for each step within this sequence. These melanoma-specific steps—across both human BM samples and intracranial/intravital models—that have been consistently validated include preferential microvascular arrest at permissive junctions, integrin-mediated endothelial interaction, chemokine-directed paracellular transmigration, and proteolytic basement membrane remodeling; after these steps, there appears to be an early reliance on vascular co-option vs. de novo angiogenesis [58]. Therefore, the above-mentioned features constitute high-confidence core events in melanoma CNS entry. On the other hand, the downstream signaling hierarchy that links particular adhesion modules to YAP/TAZ-dominant states, and the degree to which vessel-derived mediators (i.e., NO, PDGF-BB, CXCL5) are needed in a subtype-independent manner, continue to be more model- and lesion-dependent [63]. We present the above-mentioned mechanisms as plausible modifiers of perivascular adaptation but note that the causal weighting of these mechanisms in melanoma BM will require further systematic validation focused on melanoma.

After melanoma cells enter the vascular space, they attach to laminin-511 on the endothelial cells through β1-integrin, establish N-cadherin-mediated junctions, and syndecan-glypican family members of heparan sulfate proteoglycans. This attachment initiates the ILK-YAP/TAZ and RAC1-PAK signaling pathways, which link mechanical force to transcriptional programs involved in cell motility and viability [64]. Intravital microscopy has shown that the co-opted vessels are not static: they continue to secrete NO, PDGF-B, and CXCL5, which increases the adhesive properties of melanoma cells to the endothelial cells. Therefore, the dynamic reciprocal relationship between melanoma cells and endothelial cells explains why anti-angiogenic therapies are so rare in eliminating brain metastases: they do not eliminate the existing vascular network that melanoma colonizes [65]. Despite melanoma’s rapid involvement in numerous neurovascular interfaces following its invasion through the BBB, there is, at present, a considerable gap between speculation about potential mechanisms of neuro-modulation—e.g., field desynchronization at the circuit level, “editing” of perineuronal heparan sulfate charge patterns, and wide-scale regulation of all types of ion channel activity—and direct empirical support for these mechanisms in melanoma brain metastasis [12]. These ideas stem from converging lines of evidence that melanoma cells up-regulate genes related to synaptic scaffold proteins, alter perivascular matrix components, and exhibit an extensive heterogeneity of both voltage-gated and mechanically sensitive ion channels; endothelial and neuronal elements reciprocally display changes in gene expression; and spatially resolved datasets are increasingly showing adjacency patterns which suggest cross-talk between neurons and melanoma cells rather than just simple co-optation [66].

Nevertheless, it is clear that there is very little direct evidence of melanoma-specific dependence on field-responsive growth, melanoma utilization of programmable glycan ‘codes’ for access to synapses, or functional necessity for synchronized action of multiple ion channels in order to grow in MBMs [67]. Thus, these mechanisms can best be viewed as biological plausibility extensions of the initial adaptation of melanocytes with their environment rather than established selective advantages for metastasis. In order to test these hypotheses, there will need to be melanoma-focused electrophysiological experiments, targeted CRISPR disruption of melanoma-modifying enzymes that modify the extracellular matrix, high-resolution synaptomics, and in vivo experiments testing whether the modification of circuits specifically designed for MBMs will affect growth [68,69].

### 3.2. Perivascular Niche Formation and Glial–Vascular Reciprocity

Following melanoma cells’ establishment of a relationship with the vascular system, they begin to establish a perivascular niche that is similar to neural stem cell niches. Astrocytic endfeet surrounding the co-opted vessels respond to tumor-derived HMGB1, S100B, and extracellular ATP with a stimulation of NFκB-TLR4 and P2X7 signaling in astrocytes. Initially reactive, these astrocytes eventually take on a trophic function and increase expression of glutamate transporters (EAAT1/2), monocarboxylate carriers (MCT1/MCT4), and GFAP, creating a biochemical buffer zone around the metastatic cluster [70,71]. Exosome-based signaling is essential in establishing this trophic function. Exosome-derived miR-19a, miR-21, and miR-29b suppress PTEN expression and activate the PI3K-AKT-mTOR pathway in melanoma cells, thereby increasing their survival under oxidative stress. Melanoma cells produce exosomes that contain S100A4, ATP7A, and MET mRNA that modify the calcium homeostasis of astrocytes and copper metabolism, thereby supporting angiogenic remodeling [72]. Therefore, exosome trafficking represents a molecular communication that converts reactive gliosis into metabolic cooperation. Pericytes represent a third regulatory layer in the brain. Pericytes regulate BBB permeability through PDGFRβ, TGFβ-SMAD2/3, and Notch3 signaling pathways and release extracellular matrix components such as laminin-211, collagen VI, and nidogen to reinforce the vascular scaffold [73]. Spatial single-cell RNA sequencing indicates that pericytes up-regulate ANGPTL4 and SERPINE1 near melanoma cell aggregates, suggesting that they are involved in the generation of a pseudo-stem niche. Ultimately, the product of these three layers of regulation is a microenvironment that provides continuous metabolic flux: lactate produced by astrocytes and pericytes enters melanoma cells through MCT1/4, feeds the TCA cycle, and supports NAD^+^ homeostasis during hypoxia [74].

**Figure 1:**
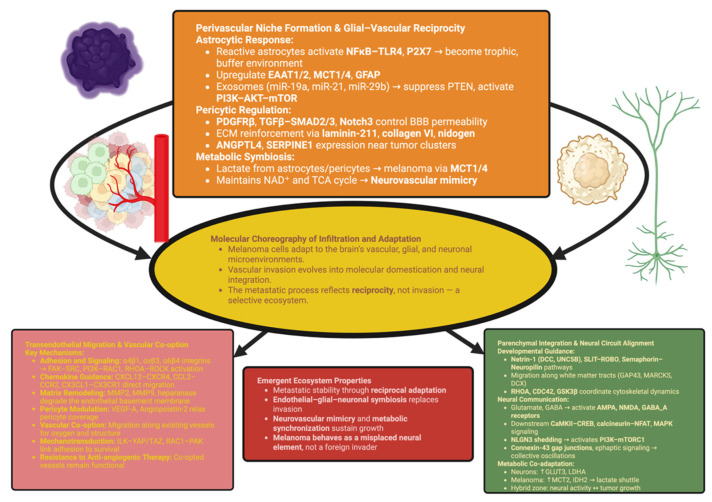
Melanoma brain metastases are formed by sequential and reciprocal adaptations that occur in three different domains: vascular, glial, and neuronal. (Left) Melanoma cells utilize α4β1, αvβ3, and α6β4 integrins, with FAK-SRC, PI3K-RAC1, and RHOA-ROCK pathways for adhesion and remodeling of the endothelium (MMP2/9 and heparanase) and migration along pre-existing vessels (resistant to anti-angiogenic therapy) during transendothelial migration and vascular co-opting. (Top) Reactive astrocytes (NFκB-TLR4 and P2X7) and pericytes (PDGFRβ, TGFβ-SMAD2/3, Notch3) form glial–vascular reciprocity within the perivascular niche. Exosomes containing miR-19a, miR-21, and miR-29b suppress PTEN and activate PI3K-AKT-mTOR. Lactate is transported between neurons and melanoma cells via MCT1/4, supporting neurovascular mimicry. (Right) Melanoma cells follow developmental cues (Netrin-DCC/UNC5B, SLIT-ROBO, semaphorin–neuropilin) to integrate into the parenchyma and align themselves with neuronal circuits. They interact with neuronal signaling via AMPA, NMDA, and GABA_A receptors, NLGN3 shedding, and connexin-43 gap junctions. These interactions lead to metabolic co-adaptation (neuronal GLUT3 → melanoma MCT2/IDH2). (Bottom) The metastatic niche created by melanoma cells functions as a stable neuro-metabolic network that maintains its function through reciprocity with the host rather than invasive processes.

Therefore, the communication between perivascular cells and melanoma cells creates a self-reinforcing metabolic symbiosis: the glycolytic activity of astrocytes, the contractile activity of pericytes, and the oxidative metabolism of melanoma cells form a closed trophic loop—the phenomenon of neurovascular mimicry. This cooperative metabolism is the first step towards adopting the organization of the brain by melanoma cells. Melanoma cells align progressively with the vascular, glial, and neuronal systems of the host, transforming from an invasive form of cancer into an active participant in the functioning of the brain’s ecosystem [75]. The diagram shown below (Figure 1) illustrates a summary of this evolutionary progression (from vascular co-option to neural integration), describing how melanoma metastasis can achieve stability when its cells are “domesticated” as part of the selection process that occurs in the brain.

### 3.3. Parenchymal Integration and Neural Circuit Alignment

In addition to interacting with the vascular compartment, melanoma cells travel through the neural parenchyma using the same molecular gradients that direct axonal growth during development. High-resolution proteomics and in situ hybridization have identified the presence of netrin-1 receptors (DCC and UNC5B), SLIT-ROBO proteins, and semaphorin–neuropilin complexes in brain metastases, therefore identifying a developmental map for the directed migration of melanoma cells [76]. Activation of the RHOA, CDC42, and GSK3β enzymes coordinates the actin–microtubule system to enable saltatory migration of melanoma cells along myelinated tracts. Protrusions of melanoma cells rich in GAP43, MARCKS, and doublecortin travel through white matter and frequently align with oligodendrocyte processes, which are physical guides for the movement of melanoma cells. At the same time, the local concentration of neurotransmitters applies selective pressures on melanoma cells to develop neural-compatible phenotypes [77]. Extracellular glutamate and GABA activate melanoma cells via AMPA, NMDA, and GABA_A receptor subunits. Activation of these receptors results in the influx of Ca^2+^ into melanoma cells, which in turn activates the CaMKII-CREB, calcineurin-NFAT, and MAPK cascades to promote metabolic adaptation and mitochondrial fusion. Mitochondria in intracerebral melanoma cells display extended morphologies and perinuclear clustering indicative of increased oxidative phosphorylation and calcium buffering similar to that observed in neurons [78,79]. Electrophysiology and optical studies have shown that melanoma cells interact with neurons to create functional synapses. The activity-dependent shedding of NLGN3 from the surface of neurons activates the PI3K-mTORC1 signaling pathway in melanoma cells and enhances their proliferative potential [80]. Connexin-43-based gap junctions enable the exchange of ions and metabolites between melanoma cells and astrocytes, while ephaptic interactions across narrow extracellular spaces allow for the transmission of low-frequency field potentials between melanoma cells and neurons. These bioelectric communications coordinate the melanoma cell clusters into partially synchronized assemblies that exhibit collective calcium oscillations similar to those present in developmental networks [81].

Lastly, spatial metabolomics show that there is a region-specificity to the metabolic coadaptation of neurons and melanoma cells; peritumoral neurons have increased expression of GLUT3 and LDHA, while adjacent melanoma cells have increased expression of MCT2 and IDH2 and utilize the latter to produce a cross-compartment lactate shuttle [82]. This bidirectional metabolic entrainment effectively eliminates the distinction between tumor and circuit and produces a hybrid zone in which neural activity regulates the kinetics of tumor growth and vice versa [83]. Based on this perspective, the brain is not just a physical site of metastatic melanoma cells but an actively engaged participant in their reeducation. Each of the various types of cells in the brain—endothelial cells, astrocytes, pericytes, and neurons—produces cues that are interpreted by melanoma cells using pre-existing developmental logic. Therefore, the resulting metastatic ecosystem is regulated by reciprocity, not invasion: the tumor interacts with, and is influenced by, the neural environment [84,85]. This dynamic equilibrium illustrates how a cancer cell can gain stability without disrupting the context of the host by adopting the design principles of the organ being colonized. Therefore, in melanoma brain metastasis, the evolutionary memory of the neural crest and the selective forces of the cerebral environment converge to produce a malignancy that operates more like a misplaced member of the brain’s network architecture rather than an invading entity [86].

## 4. Synaptic Mimicry and Electrophysiological Coupling

Following their stabilization in the vascular and glial environments, melanoma cells undergo progressive structural and functional alterations that enable their interaction with the neural environment. This includes electrophysiological and synaptic mimicry, where melanoma cells interact with neurons, using molecular mechanisms similar to those used by neurons for communication. The molecular junctions, ion channel repertoires, vesicular machinery, and rhythmic activity patterns enable melanoma cells to be part of local information flows in the CNS [87,88].

### 4.1. Molecular Architecture of Melanoma–Neuron Junctions

Melanoma cells establish defined contact zones with neurons that resemble excitatory and/or inhibitory synapses but remain malignant entities. The molecular organization of these contact zones involves the neuroligins/neurexins, Slitrk’s, LRRTMs, and other synaptic organizing molecules, to cluster AMPA, NMDA, or GABA receptor subunits on opposite sides of the cell membrane [89,90]. The structure of these contact zones is influenced by local variations in heparan-sulfate patterns and thereby the ability of neurexins to bind their ligands and thus melanoma–axon or melanoma–dendrite contact specificity. The geometric characteristics of the junction are further influenced by the presence of ephrins, which regulate the cytoskeleton of melanoma cells by acting through Rho GTPase-dependent mechanisms, maintaining the necessary nanoscale spatial arrangement for ephaptic interactions [91,92].

Furthermore, melanoma cells have an efficient synaptic vesicle cycle, using the SNARE proteins (syntaxin-1A, SNAP25, VAMP2) and UNC13/Munc13 to prime and subsequently exocytose synaptic vesicles. After the vesicle has been exocytosed, it is retrieved from the surface by a dynamin/clathrin-dependent mechanism [93]. Individual melanoma projections express primarily glutamatergic or GABAergic receptors and thus can sample local tonic activity [94].

Astrocyte-derived factors such as thrombospondin-1/2, hevin, and glypican-4/6 contribute to the maturation of these junctions, while the perisynaptic ECM components (aggrecan, brevican, hyaluronan) accumulate around the tumor–neuron interface. Furthermore, melanoma-secreted metalloproteases remodel the perisynaptic matrix to control glutamate and potassium diffusion [95,96].

Together, the above elements create hybrid junctions that utilize common synaptic proteins to mediate communication while providing a direct link to oncogenic pathways regulating melanoma cell migration, metabolism, and survival [50].

### 4.2. Bioelectric Coupling and Network Synchronization

Melanoma cells exhibit bioelectric behaviors generated by their position relative to regional glutamate levels, their membrane conductivity, and the mechanical forces they experience. Using patch-clamp and voltage-sensitive dyes, subpopulations of melanoma cells have been identified that differ in terms of their resting potential, with some subpopulations having more depolarized resting potentials in areas of higher glutamate levels [97]. HCN and KCNQ channels produce sub-threshold resonance in melanoma cells, while T-type and L-type Ca^2+^ channels induce low-frequency oscillations in melanoma cells that result in the activation of CREB and NFAT. Plateau phases in melanoma cells are produced by STIM1/ORAI1 store-operated Ca^2+^ entry and mechanosensitive TRPM7/TRPV4 channels, which transform perivascular forces and osmotic conditions into electrical signals [98].

Electrical integration of melanomas occurs via multiple pathways:

- Gap junction networks based on connexin. Connexin-43/30 heterotypic channels enable melanoma cells to communicate with astrocytes and establish equilibrium of local potassium and distribute second messengers (IP_3_ and cAMP) [99].
- Ephaptic microdomains. Tight juxtaposition of melanoma cell membranes to adjacent axon membranes facilitates contactless current transfer. Synchronous firing of neighboring axons imparts low-frequency potentials to melanoma membranes and thus synchronizes the timing of Ca^2+^ spikes [12].
- Purinergic lattices. Release of ATP from neurons and astrocytes activates P2X4/P2X7 on melanoma cells, generating rapid inward currents that regulate vesicle fusion and signaling related to the inflammasome; activation of metabotropic P2Y receptors regulates longer-term plasticity via activation of PLC-DAG-IP_3_ pathways [100].
- Adrenergic/cholinergic modulation. α7-nAChR microdomains on melanoma cells produce Ca^2+^ microtransients with high fidelity to cholinergic tone; β-adrenergic inputs regulate the PKA-EPAC axes that control the metabolic and electrical states of melanoma cells during arousal cycles [101].

Mitochondrial dynamics regulate these signals: Ca^2+^ handling at ER–mitochondria contacts (MCU-MICU1) and the regulation of mitochondrial cristae by OPA1 sustain oxidative phosphorylation, while DRP1-mediated fission supports oscillatory competence [7].

At the population level, multi-electrode array and two-photon Ca^2+^ imaging studies have shown that melanoma cell populations are synchronized with nearby neurons [102,103]. The synchronization of melanoma cell populations with nearby neurons is stabilized by the astrocytic expression of Kir4.1 and aquaporin-4 by controlling extracellular K^+^ and water fluxes. Local GABA metabolism modulates the firing probability of melanoma cells and demonstrates that the inhibitory/excitatory balance also exists in the tumor compartment [104].

### 4.3. Information Flow, Plasticity, and Circuit-Level Consequences

Melanoma cells can both read and write local circuit activity. Glutamate, ATP, etc., are released from melanoma cells by Ca^2+^-dependent vesicle release, and these substances modify the dendritic integration threshold of neurons and contribute to short-range facilitation loops [105,106]. Apposition of melanoma cells to the somata of interneurons reduces the inhibitory gain of these interneurons, thereby reducing the microcircuit resonance without generating seizure activity. Furthermore, melanoma projections aligned along axons in white matter influence conduction reliability by controlling the potassium and lactate concentrations in the periaxonal space [107].

The hybrid junctions demonstrated synaptic-like plasticity: coincident melanoma–neuron activity increases the strength of the connections by increasing the amount of AMPAR inserted into the membrane via CaMKII and stargazin-dependent mechanisms, whereas activity that occurs asynchronously weakens connections via calcineurin-dependent mechanisms [108]. Tumor–tumor connections over long distances occur via tunneling nanotubes (TNTs), which transport mitochondria, Ca^2+^ waves, and synaptic proteins; TNT-connected nodes show phase coherence over millimeter distances and thus form a metastatic syncytium [109]. The mechanical properties of the perisynaptic matrix influence the oscillatory behavior of melanoma cells, as LOX-mediated cross-linking provides stiffness-dependent tuning of frequency and amplitude, and thereby influences whether melanoma cells will migrate or proliferate [110]. Micro-glial C1q/C3-dependent pruning of weak tumor–neuron connections while preserving strong and metabolically active connections is obtained [111,112].

Feedforward (neuron → melanoma → astrocyte) and feedback (melanoma → interneuron → neuron) motifs have been identified using spatial transcriptomics and Ca^2+^ imaging that help maintain local circuit stability. These motifs have redundancy, and disrupting one connection (i.e., blocking MCT2 or connexin-43) typically leads to compensatory rewiring instead of loss of function, and this explains why most single-agent therapies in the CNS show little effectiveness [113]. Table 2 provides a sequence of how melanoma progresses (structurally) as a single cell infiltrate to (electrically) become part of neural circuits. The table aims to represent the molecular, cellular, and electrical processes involved in creating the structural and functional changes that occur in melanoma brain metastases: those involved in the creation of synaptic mimicry and resulting neuroadaptive behaviors.

#### Synthesis

Synaptic mimicry of melanoma cells represents not a superficial phenomenon but rather a functional incorporation of design principles of neural systems into the biology of malignancy. Adhesion molecules specify the detailed nano-architecture of the junction; vesicle cycles and receptor scaffoldings translate the electrical context into biochemical responses; gap junctions, ephaptic fields, and purinergic lattices extend the communication beyond ligand diffusion; mitochondria, channels, and matrix interact to couple energy, mechanics, and information [119]. Hybrid motifs exist at the circuit level that preserve local stability while supporting the continued existence of tumors. Rather than duplication of the synapse, this represents the adaptation of its logic—a convergence of where malignancy has learned to communicate and to modestly edit the electrical language of the brain [120].

## 5. Metabolic Reprogramming and Neuroenergetic Integration

Neural tissues provide a highly competitive environment for tumors to survive. As melanoma develops electrochemical and synaptic coherence with the surrounding neural tissue, so too do its metabolic processes undergo significant changes. The changes in melanoma’s metabolism are not just secondary adaptations; they are necessary to enable the melanoma to exist in the tight metabolic margin of the brain [121].

The brain has one of the smallest physiological metabolic margins; deviations in ATP, glucose, and redox homeostasis can quickly disrupt neurotransmission, ionic balance, and cell viability. For melanoma to survive in this environment, it must be able to survive fluctuations in oxygen and nutrient availability and must be able to join the neuroenergetic economy of the brain. It will need to share metabolic substrates, redox signals, and mitochondrial dynamics with neurons and glia [122,123]. Section 5 describes this dialog and how melanoma transforms classic tumor metabolism to be compatible with the metabolism of neurons.

### 5.1. Reversal of the Warburg Paradigm in the Brain Microenvironment

Unlike many brain metastases that use aerobic glycolysis, melanoma in the brain uses a metabolic reversal due to its proximity to oxygen-rich lactate-producing glial and vascular cells. Instead of exporting lactate, melanoma in the brain is a lactate consumer. Glial and vascular cells in the brain produce lactate through glycolysis and shuttle it through MCT1 and MCT4 to melanoma. In turn, melanoma expresses MCT2, LDHB, and IDH2 to take up lactate and convert it to pyruvate to be used in the TCA cycle. This adaptation enables melanoma to continue to perform oxidative phosphorylation (OXPHOS), even under conditions of hypoxia, thus preserving glucose for neurons and limiting metabolic competition [124,125]. Studies have shown that the mitochondria of melanoma exhibit an increase in cristae surface area, enhanced OPA1-mediated mitochondrial fusion, and an increase in the expression of respiratory chain complex I and IV. The resulting high-efficiency OXPHOS enables prolonged electrical signaling and vesicle cycling at low levels of reactive oxygen species (ROS). The switch from glycolytic flux to oxidative metabolism is regulated by PGC1α, NRF2, and MITF at the level of transcription and reinforced by epigenetic demethylation of the promoters of genes involved in mitochondrial biogenesis [126].

At the same time, there is not an equal amount of reverse-Warburg/OXPHOS leaning across all melanoma brain metastasis. Human and model data also indicate a continuum of metabolic states within the intracranium; however, most brain metastases are characterized by a high rate of lactate utilization and an increased mitochondrial content, while some remain primarily glycolytic; some lesions have adopted hybrid glycolytic–OXPHOS pathways; and some have adapted to low-oxygen environments due to poor perfusion in certain areas of the tumor [19,127]. The variation among these brain metastases likely represents different degrees of blood supply proximity, oxygen tensions, clonal lineages, and treatments prior to the development of the brain metastasis [128]. Therefore, we consider reverse-Warburg metabolism to be a common method for melanoma brain metastases to establish an energy-compatible relationship with the neural environment, although not required nor universal in melanoma brain metastases.

Thus, the reverse-Warburg effect causes the energy metabolism of melanoma to become aligned with the oxidative hierarchy of the brain. Neurons use glucose and lactate dynamically to meet their energy needs, astrocytes act as a buffer and supplier of these energy sources, and melanoma acts as a third oxidative node that is capable of using both energy sources and acting as a buffer for the redox potential of the brain [129].

### 5.2. Mitochondrial Networking and Redox Coupling

Melanoma mitochondria do not reside in isolation. They participate in a dynamic continuum of metabolic and redox interactions with nearby cells. Electron microscopy and live-cell imaging demonstrate that melanoma mitochondria form contact sites with the endoplasmic reticulum (ER), not only within melanoma cells but also transiently with ER structures of nearby astrocytes and endothelial cells [130,131]. These contact sites facilitate coordination of Ca^2+^ flow, lipid transfer, and ROS signaling and result in coordinated metabolic oscillations between the tumor and glia. TNTs allow for the direct transfer of mitochondria from one cell to another. During periods of oxidative stress, astrocytes transfer intact mitochondria to melanoma cells, and this occurs through the action of Miro1 and Mitofusin-2 [132]. This transfer restores the bioenergetic capacity of melanoma cells after exposure to ionizing radiation or anti-PD-1 therapy. Conversely, melanoma cells export defective mitochondria through the same TNTs as those used for mitochondrial transfer, and this results in a reduction in the oxidative load of melanoma cells and an imposition of a metabolic burden on nearby glia cells, similar to that seen in the outsourcing of mitophagy to glia cells seen in some neurodegenerative diseases [133,134].

Glutathione cycling, thioredoxin, and NADPH systems are present in melanoma cells and contribute to maintaining the redox state of these cells. Additionally, melanoma cells have employed a novel means of regulating redox states, i.e., through the transfer of redox equivalents to nearby cells. Specifically, gap junctions composed of connexin-43 enable the diffusion of reduced glutathione and other small thiols between melanoma and astrocyte cells [135,136]. This low-amplitude redox synchronization limits oxidative stress signaling and maintains the integrity of neuronal circuits. Furthermore, redox signals can influence gene expression. Oscillating pulses of hydrogen peroxide can stimulate NRF2–ARE pathways and modify the expression of antioxidant and metabolic genes in both melanoma cells and nearby astrocytes. Therefore, redox signals can serve as a cross-compartmental second messenger that promotes a cooperative oxidative equilibrium in the tumor–glial interface rather than serving solely as a signal of oxidative damage [137].

### 5.3. Neurotransmitter-Derived and Ion-Dependent Metabolism

Metabolism of melanoma in the brain involves more than just the classical substrates, such as glucose and glutamine, but also includes the products of neurotransmitter release and the ion gradients generated by the activity of neurons. Extracellular glutamate and aspartate, produced by neuronal activity, are taken up by melanoma cells via the transporters EAAT1/2 and SLC1A3 and are converted into TCA cycle intermediates via the action of transaminases (GOT1/2) [138]. This enables melanoma cells to link their rate of proliferation to the rate of synaptic activity. Simultaneously, GABA shunt activity is expressed as an additional anaplerotic pathway. Expression of GAD1, ABAT, and SSADH enables melanoma cells to convert GABA into succinate, linking neuronal inhibition with respiration in the tumor. The presence of a GABA–succinate axis indicates that this represents a conserved metabolic loop that was originally established during development of the CNS, where the production of energy and the cycling of neurotransmitters were coupled [139].

Ion gradients generated by the activity of neurons can also modulate energy metabolism. Intracellular calcium oscillations in melanoma cells can control mitochondrial respiration via the action of CaMKII–PGC1α. Furthermore, chloride flux through NKCC1/KCC2 transporters can influence cellular volume and ATP consumption. Elevated intracellular sodium generated by sustained depolarization can stimulate Na^+^/K^+^-ATPase and thereby establish a relationship between membrane activity and mitochondrial workload [140]. This relationship is stabilized by the action of AMPK sensing and adaptation through the actions of LKB1 and SIRT3 signaling. Thus, the electrochemical and metabolic domains of melanoma cells are linked to each other and to the activity of the neural networks in which they reside [141].

### 5.4. Lipid, Copper, and One-Carbon Metabolism in Neural Adaptation

The lipid environment of the brain requires specialized metabolic processing. Melanoma cells adapt to this environment by expressing FABP7, CPT1A, and SCD1, thereby facilitating the transport and oxidation of long-chain fatty acids found in abundance in myelinated tracts. Oxidation of fatty acids generates NADH during periods of inactivity of neurons, whereas lipid droplets can act as transient reservoirs for reactive aldehydes [142]. Copper metabolism in melanoma is dramatically different from that of melanoma growing outside the brain. Melanoma cells express ATP7A and CTR1 to regulate copper transport and the efficiency of electron transport through cytochrome c oxidase. This adaptation is important in the copper-rich environment of the synaptic cleft and contributes to both angiogenic remodeling and the regulation of synaptic enzymes [143].

**Figure 2:**
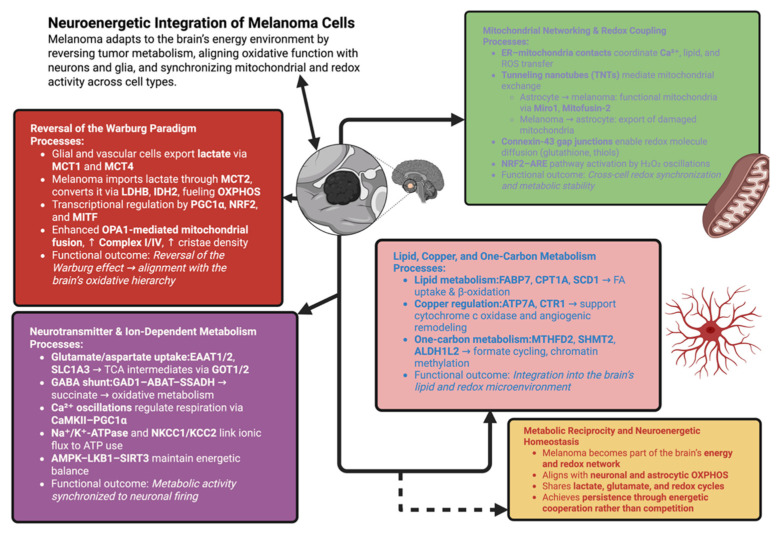
Illustration of how melanoma cells adapt to a brain’s limited metabolism using progressive neuroenergetic integration. Here is an illustration of melanoma cells consuming lactate from glial and vascular cells through MCT2 and performing oxidative phosphorylation (under the control of PGC1α, NRF2, and MITF) as a reversal of the Warburg effect.

Finally, the one-carbon metabolic network in melanoma cells consists of MTHFD2, SHMT2, and ALDH1L2, providing both redox buffering and precursors for nucleotide synthesis and participating in the folate cycles of neurons. Transfer of formate between melanoma cells and astrocytes maintains methylation potential in hypoxic environments and supplies histone methyltransferases with substrates to support chromatin organization and the integration of metabolic status and chromatin structure [144]. Rather than competing with neurons and glial cells for glucose, melanoma cells in the brain are able to coordinate with them by synchronizing their own metabolism with that of the surrounding neural tissue and integrating into its oxidative and redox networks [145]. This is depicted in Figure 2, where a reversal of glycolysis and a network of mitochondria can be seen as transitioning into cooperative interactions dependent on neurotransmitters and lipids, thereby allowing melanomas to survive via metabolic coordination rather than competition.

#### Synthesis

Melanoma cells in the brain have evolved to be more than just cancerous cells; they have become part of the neuroenergetic system of the brain. Melanoma cells in the brain have inverted the Warburg effect, import lactate and glutamate, synchronize mitochondrial rhythmicity with astrocytes and neurons, and maintain common redox and ionic homeostasis with these cells [59]. This integration does not stabilize malignant growth; it stabilizes growth within the logic and homeostatic mechanisms of the brain, producing a state of equilibrium rather than conflict. Melanoma cells achieve persistence in the brain by becoming part of the same energetic, rhythmic, and reciprocal logic of the neural system [90].

## 6. Immune Cross-Talk and Neuro-Immune Modulation

While melanoma’s metabolic and electrophysiological adaptation in the brain contributes to its coexistence with the brain, it has created a new immunological environment in the brain. The brain is not simply an “immune desert,” but an area of selective permissivity. Inflammation and immune tolerance exist in a delicate balance in order to protect the neural function [146]. Section 6 intends to show how melanoma communicates using the biochemical language of neuro-immunology and transforms classic forms of immune evasion into immunological mimicry; and how melanoma exploits the molecular grammar of neuro-immune signals to reprogram micro-glial and astrocytic cells to be protective partners and establish a long-term equilibrium between the immune system’s ability to survey the tumor and the need to preserve the neural tissue.

### 6.1. The Immunological Architecture of the Brain

There are two key principles that shape the brain’s immune structure: the prevention of inflammation without harming the brain and the prevention of immune tolerance without neglecting the brain. An ecosystem consisting of micro-glial cells, astrocytes, perivascular macrophages, and meningeal lymphocytes was developed to respond to the brain in a precise manner while minimizing damage to the brain [147]. Due to the compartmentalization of cytokine responses and the tight regulation of antigen presentation, the risk of damaging neurons during inflammation is minimized. Therefore, the success of melanoma in the brain depends on melanoma adapting to the brain’s immune architecture, and not fighting it [148].

When melanoma first enters the brain, it encounters an environment dominated by micro-glial cells, which express the receptors TREM2, CX3CR1, and complement receptors. Initial contact between melanoma and micro-glial cells is initiated through the recognition of melanoma by micro-glial cells through TLR2/4 and STING pathways, which lead to a transient pro-inflammatory response. The response consists of the release of IL-1β, TNF-α, and type I interferons [59]. Melanoma responds to this pro-inflammatory response by releasing suppressive mediators including CSF1, TGF-β, and CCL2, which reprogram micro-glial cells to become trophic, anti-inflammatory cells characterized by increased expression of Arg1, IL-10, and PD-L1. Astrocytes also respond to melanoma by activating NF-κB–TLR4 and P2X7 pathways when exposed to tumor-derived HMGB1, S100B, and ATP [149]. As a result of the activation of these pathways, there is transient reactive gliosis. Over time, the signaling through STAT3, ERK1/2, and SOCS3 results in the conversion of astrocytes to a repair cell-type characterized by increased expression of MCT1/4, VEGF-A, and CXCL12. Therefore, what initially begins as an innate response to melanoma ultimately results in a symbiotic relationship between the brain’s immune cells and melanoma, favoring stability versus eradication [150,151].

### 6.2. Synaptic Immunity and Checkpoint Signaling

Melanoma uses checkpoint molecules in the brain that were originally used to regulate synaptic transmission to create a barrier to immune attacks. The use of checkpoint molecules by melanoma involves the abnormal expression of molecules that are typically found at inhibitory synapses on melanoma cells and in tumor-derived exosomes. Examples include neuroligin–neurexin pairs, GABA_A receptor subunits, and neuropilin-2 [152]. These molecules mimic synaptic “off” switches and create inhibitory synapse-like interactions with cytotoxic T cells and micro-glial cells. Neuroligin-1 interaction with neurexin-like ligand on T-cells decreases calcium ion flux and cytokine release, thus decreasing immune activation. The expression of GABA_A receptor alpha2/beta3 subunits by melanoma cells decreases local chloride ion balance and membrane potential, thereby decreasing the formation of the immune synapse. Neuropilin-2, which is typically involved in axonal guidance, can bind semaphorins on micro-glial cells and suppresses the movement and antigen-presenting capabilities of these cells [153].

The boundaries of evidence for this immune evasion pattern at the level of the synapse, when framed under the synaptic–immunity paradigm: In terms of the strengths of evidence supporting the immune evasion landscape seen in melanoma brain metastases, there are clear differences [154]. The canonical pathways (PD-L1/PD-1 pathway, the broader repertoire of checkpoint molecules active in melanoma brain metastasis, etc.) involved in the immune tolerance observed in melanoma BM have been repeatedly demonstrated to exist in human samples and specific in vitro/in vivo melanoma models of the brain and thus can be considered as having high confidence as causal contributors to CNS immune suppression [155]. On the other hand, the synaptic-parallel molecules mentioned above have not yet been directly shown to modulate immunity in melanoma in vivo but instead have been identified based on their expression, spatial association, presence in tumor-derived vesicles, and functional impacts in organotypic or co-culture systems [156]. Therefore, we believe that synaptic immunity likely represents an additional modulatory layer that could either augment or fine-tune previously well-established checkpoint and micro-glial programs in melanoma, rather than an equally valid immune modulatory mechanism in and of itself.

Checkpoint molecules, such as PD-L1, Galectin-9, and CD200, are expressed in response to synaptic activity and beta-adrenergic stimulation. The expression of these checkpoint molecules reflects a cross-talk between neural activity and immune suppression. This dynamic responsiveness is consistent with the observation that immune checkpoint blockade (ICB) therapies have variable effects in treating brain metastases: the effect of ICB therapies depends on the local microenvironment and therefore relates to neuronal activity and immune suppression [103,157].

### 6.3. Micro-Glial and Astrocytic Reprogramming

Micro-glial cells and astrocytes represent a partnership that provides support to melanoma growth and persistence in the brain. Spatial transcriptomics analysis of micro-glial cells near melanoma demonstrates that there is a homeostatic-to-regenerative transition in micro-glial cells that is associated with increased levels of C3, IL-10, and TREM2 and reduced levels of CXCL10 and MHC-II. The functional role of these micro-glial cells is to participate in complement-mediated removal of weak synaptic connections in proximity to tumor margins, which reduces excitotoxicity and indirectly limits the access of T-cells to the tumor [158]. Melanoma actively promotes this transition by delivering miR-21, miR-29b, and miR-146a via exosomes that target NF-kappa B and IRF pathways in micro-glial cells and thereby suppress the expression of inflammatory genes. In addition, melanoma delivers CSF1 and IL-34 to micro-glial cells, which activates micro-glial CSF1R and thereby expands a population of trophic/phagocytic micro-glial cells that remodel the extracellular matrix and maintain a non-inflammatory phenotype [159].

Astrocytes contribute to this process by reciprocal metabolic and signaling relationships with melanoma. The exposure of astrocytes to melanoma-derived VEGF, CXCL12, and WNT7A induces astrocyte expression of SERPINE1, ANGPTL4, and TGF-beta2, which stabilize blood vessels and reduce immune activation. Reactive astrocytes surround melanoma aggregates forming an anatomical barrier that is physiologically supportive, enriching the peritumoral region with glutamate transporters and monocarboxylate transporters that provide the tumor with energy through oxidative phosphorylation. This astrocytic participation is not simply defensive but is a form of immunological containment that transforms inflammation into metabolic support [160].

### 6.4. Peripheral–Central Immune Interfaces

Even though melanoma resides in the brain’s “immune sanctuary,” melanoma still interacts with the peripheral immune system through meningeal lymphatic conduits and the choroid plexus gateway. Tumor antigens drain through the glymphatic–meningeal axis and reach cervical lymph nodes where they undergo antigen presentation. However, the antigenic imprinting in this pathway typically leads to the generation of regulatory T-cells (Tregs) due to soluble IL-33 and TGF-beta released from astrocytes and endothelial cells [161]. These Tregs then migrate back to the brain, directed by CXCL12/CXCR4 signaling, and secrete IL-10 and adenosine, promoting local immune tolerance. Peripherally derived macrophages that migrate into the CNS develop into hybrid macrophages that are located adjacent to co-opted vessels and contribute to immune privilege and regulate microvascular tone through PDGFRbeta and endothelin-1 signaling [162,163].

Intracranially administered ICB therapies transiently disrupt this equilibrium. Anti-PD-1 or anti-CTLA-4 treatment may allow effector T-cells to migrate into the CNS; however, melanoma rapidly reverses this by increasing IDO1, AHR, and GABAergic signaling to re-establish metabolic and immunologic homeostasis. The cycling nature of this resistance pattern indicates that intracranial immune control is maintained through a series of neuro-immune feedback loops, rather than through a permanent suppression mechanism [164].

### 6.5. Neurotransmitter Modulation of Immunity

Increasing evidence suggests that neurotransmitters act as immune modulators in the metastatic niche.

- Glutamate acts through mGluR3 and mGluR5 on micro-glial cells to decrease phagocytic activity and cytokine production, establishing an anti-inflammatory environment [165].
- Norepinephrine and dopamine, present in perivascular areas, interact with D2 and beta2-adrenergic receptors on immune cells to decrease antigen presentation and cytotoxic responses [166].
- Serotonin (5-HT) acting through 5-HT2A/1B regulates the release of cytokines and the activity of micro-glial cells, resulting in lower sensitivity to immune stimuli during periods of neural activity [167].

Melanoma interacts with the immune system through the expression of receptors for these neurotransmitters and the synthesis of the neurotransmitter ligands themselves by TPH1 or TH. This enables the tumor to modulate the immune tone in real time based on neuronal activity and sleep–wake cycles, creating a neuro-immune entrainment that further stabilizes coexistence [168].

#### Synthesis

The immune environment in melanoma brain metastases is not solely about escaping immune surveillance but rather integrating and modifying it. Through the mimicking of synaptic inhibition, the reprogramming of micro-glial and astrocytic cells, and the association of immune checkpoints with neural activity, melanoma transforms passive immune tolerance into a dynamic, active equilibrium [169]. The resulting state resembles a homeostasis of immunity, in which surveillance is suppressed but not eliminated, and inflammation is controlled but remains metabolically productive, allowing the tumor to exist in a semi-permissible manner in the brain’s larger immune rhythm. Melanoma does not suppress the immune system but instead tunes it to match the physiology of the host organ. The neuro-immune symbiosis is one of the most complex examples of malignancy, adapting not to fight the host but to fit within the host’s own logic, and converting the brain’s protective mechanisms into a means to achieve coexistence [170].

## 7. Network Thermodynamics and the Physics of Cognitive Stability

Brain metastasis is an attractive opportunity to study melanoma’s adaptation to the neural environment, since the mechanisms supporting melanoma’s survival within the brain would necessarily require a precise synchronization of melanoma and the brain, based on three fundamental areas: development, metabolism and physiology, and immunology. These three areas of synchronization are continuously being adjusted and balanced, which creates an interdependence that supports melanoma’s survival but also defines potential vulnerabilities [171]. Section 7 will aim to explore how precision-based attacks on melanoma’s embedded neural circuits can restore the physical isolation of melanoma from its host while maintaining the normal functioning of the brain.

### 7.1. Targeting the Neuro-Melanocytic Interface

Metastasis occurs when melanoma cells interact with existing neural and vascular tissues in the brain. Previous studies have demonstrated that melanoma cells use pre-existing vascular structures rather than forming new vasculature, and therefore, anti-angiogenic treatments have failed to prevent the establishment of metastatic lesions. Instead, the focus should be to physically isolate the melanoma from its existing structural relationships [172,173].

The mechanisms by which to disrupt interfaces between the tumor and host are presented as hypotheses based on a variety of molecular, spatial, metabolic and bioelectric features of melanoma brain metastases and as preliminary data from selected pre-clinical platforms [174]. However, we have no evidence of the critical melanoma-specific in vivo evidence required for each of these potential therapeutic targets to progress towards a clinical trial, i.e., in vivo proof-of-concept, dose-limiting toxicity within the central nervous system, and reproducible efficacy across different types of heterogenous intracranial tumors [175,176]. Therefore, we outline the proposed strategies as an experimental roadmap to decouple the tumor–host interaction at the neuro-melanocytic interface: high plausibility (adhesion/mecanotransduction and perivascular guidance) and lower plausibility (synaptic adjacent trophic signaling and perineuronal net glycan architectures) are both outlined as being testable, while acknowledging that they will require specific melanoma BM models to establish their feasibility and clinical relevance in timely translational trials.

Pre-existing peptides that block integrin and mechanotransduction receptors (alpha V beta 3/beta 1) can disrupt melanoma cell migration at sites of focal adhesion and cytoskeletal polarization. Similarly, selective blockade of EphA2 or EphB4 signaling can disrupt bidirectional contact guidance and break up the perivascular niche by disrupting ephrin-Eph interactions [177]. NLGN3 represents another potential therapeutic target. NLGN3 is a trophic factor for melanoma cells that is shed from neurons through the action of ADAM10/17. Inhibiting NLGN3 shedding using inhibitors of ADAM10/17 resulted in the loss of PI3K-mTORC1 signaling and halted growth in the brain. Engineered ligands that bind to neurexins and do not activate subsequent pathways may also be used as “synaptic decouplers” (i.e., to treat the melanoma cells to inhibit cellular communication) [178].

Another target is the heparan sulfate code present on perineuronal nets. Therapeutic modification of sulfotransferase enzymes or synthetic analogs of modified glycosaminoglycans may restore selective insulation of neurons and block melanoma cell processes that take advantage of changes in charge and binding affinity to gain access to synapses [179]. While NLGN3 and neuronal activity-related factors are promising therapeutic targets, their current mechanistic understanding mostly comes from glioma/neuro-oncology and not from melanoma-specific experimental studies. In gliomas, NLGN3 is a powerful mitogen that activates PI3K–mTORC1, facilitates synaptic reorganization, and speeds electrical incorporation of tumor cells into neural networks [180]. By contrast, in melanoma brain metastases, direct evidence for the role of NLGN3 remains mostly indirect: genomic datasets indicate that synaptic scaffold genes are overexpressed; ex vivo co-cultures of melanoma cells with neurons have shown some response to neuronal stimulation; and perivascular melanoma cells exhibit structural characteristics indicative of neural mimetic behaviors. As such, while there are some data supporting functional synapse formation between melanoma cells and neurons, bidirectional electrochemical communication between them, and NLGN3-driven proliferation in melanoma, there is still very little direct evidence [181]. Therefore, we view the interaction between melanocytes and the neural environment as an example of convergent adaptations in which melanoma cells take advantage of the environmental cues derived from the nervous system, but do so without developing a full glioma-like program of synaptic organization [182]. The above distinctions have therapeutic relevance: (i) inhibiting ADAM10/17 and designing neurexin–ligand-binding molecules will likely decrease the degree of support provided by the neural environment to melanoma cells but (ii) will likely act on a broader axis of neuron-derived trophic signals rather than directly acting on a neuron–melanoma synapse [183]. Future studies using single-cell electrophysiological assays combined with spatial multi-omics and high-resolution synaptomics, along with melanoma-specific models of NLGN3 dysfunction, will be critical to determining if these interactions represent a dependence of melanoma cells upon synapses or merely an epiphenomenon associated with metastasis [184].

### 7.2. Modulation of Electro-Metabolic Coupling

Melanoma’s ability to communicate electrochemically and metabolically with the brain allows it to “talk” a common language with its host; however, this language can be therapeutically disrupted. Central to this concept is functional desynchronization—the disruption of melanoma’s electrochemical and metabolic oscillations so that they are out of sync with those of its host neural and glial networks. Blocking Ca^2+^ channel activity prevents melanoma cells from oscillating in synchrony with their host and disrupts the subsequent CaMKII-CREB transcriptional loops. Inhibiting either HCN or KCNQ channels blocks sub-threshold resonance and thus disrupts the collective signaling required for synchronized behavior [185]. Monocarboxylate transporter (MCT) inhibitors prevent lactate and pyruvate exchange between astrocytes and melanoma cells and break the neuroenergetic network. Additionally, AQP4 antagonists and LOX inhibitors disrupt the ionic and mechanical synchrony and thus destabilize both water flux and matrix stiffness required for oscillatory integrity [186].

Collectively, these disruptions create a controlled-phase chaotic condition, an asynchronous state where the rhythmic reinforcement of melanoma is removed, thereby shifting the melanoma towards oxidative imbalance and increasing susceptibility to oxidative and immunological stress. This approach is similar to cardiac anti-arrhythmic therapy—instead of stopping the heartbeat, it corrects the rhythm [146].

### 7.3. Reawakening Immunity Through Neuro-Synaptic Checkpoint Control

Immune suppression in the brain is not a passive event; it is a neurally regulated one. Therefore, the activation of the host immune system must be accomplished by the modulation of neural inputs that control checkpoint dynamics. The new therapeutic paradigm is the decoupling of immune tolerance from neural tone. Neuro-immune hybrid therapies are combinations of ICB with neuromodulatory agents. For example, the administration of β-adrenergic antagonists (e.g., propranolol) or GABA_A receptor inhibitors can temporarily decouple synaptic inhibition from immune quiescence, thereby increasing the effectiveness of ICB without inducing neuroinflammation [187].

Exosomes secreted by melanoma cells that contain neuroligin, GABA_A subunits, and neuropilin-2 are soluble checkpoints that can suppress the immune response in the vicinity of the peritumoral region. The reduction in exosome secretion (using a neutral sphingomyelinase inhibitor such as GW4869) or uptake (using heparan sulfate mimetics) may similarly diminish immune suppression. Additionally, the administration of AHR and IDO1 inhibitors can abolish tryptophan catabolism, thereby allowing effector T-cells to regain functionality and reducing the serotonergic immunosuppressive loop that synchronizes tumor and host immunity to circadian neurotransmission [188]. Reprogramming of microglia through the CSF1R blockade or TLR9 agonists restores antigen presentation while maintaining the intact anatomy of the surrounding tissue. Similarly, inhibition of STAT3 in astrocytes (using napabucasin or WP1066) disrupts metabolic cooperation and results in the transformation of astrocytes back to an antiviral-like state. Rather than stimulating the host immune system through force, therapy will stimulate the reawakening of the host immune system through disconnection—releasing the brake on the host immune system while respecting the neural environment [189].

### 7.4. Synthetic Vulnerabilities in Mitochondrial and Redox Networks

Melanoma’s mitochondrial networks are another example of the dual nature of melanoma’s dependency on the neural environment. While providing melanoma with enhanced resistance to environmental stresses, melanoma’s reliance on synchronized mitochondrial dynamics, Ca^2+^ signaling, and redox balance creates multiple opportunities for precision-based disassembly [190].

Blocking Miro1 and Mitofusin-2 halts TNT-mediated mitochondrial transfer and thus isolates melanoma metabolically. Selective inhibition of Complex I (IACS-010759) or succinate dehydrogenase-FAD coupling preferentially disables OXPHOS-dependent melanoma cells while leaving intact glycolytic glia. Blocking thioredoxin reductase or glutathione peroxidase disrupts redox homeostasis and transforms controlled ROS pulses into prolonged oxidative stress, leading to a collapse of NRF2-mediated resilience [191]. Simultaneously, blocking connexin-43 prevents diffusion of reduced thiols and glutathione between tumor and astrocytes and thus increases oxidative damage to melanoma microclusters. Future technologies, including redox-active nanoparticles, light-activated mitochondrial disruptors, and precision photodynamic systems, can build upon these dependencies with spatial specificity and ensure that the intervention remains localized to the tumor’s network nodes while maintaining the integrity of the host’s neuronal networks [192].

### 7.5. Circuit-Level and Chronobiological Interventions

As melanoma brain metastases function as a distributed neural network, therapy must advance beyond single-target inhibition to include circuit-based modification. The tumor does not exist independently; it is a dynamic element of the brain’s oscillatory economy [193,194]. Low-intensity tACS (transcranial alternating current stimulation) or focused ultrasound can selectively disrupt field synchrony in peritumoral regions and disrupt ephaptic coherence and metabolic coupling. Similarly, chronotherapy, i.e., administering immunotherapy or metabolic drugs in synchrony with the circadian rhythms of neurotransmitter release, immune surveillance, and blood–brain barrier permeability, has shown initial promise in restoring periodic vulnerabilities. The administration of ICB or metabolic inhibitors during the host’s periods of rest will increase penetration and decrease the risk of excitotoxicity [195]. Future approaches will likely employ closed-loop systems that utilize real-time electrophysiological monitoring to dynamically adjust the dosage of neuroactive or metabolic modulators. In this way, the metastasis can be treated as a network disorder—stabilizing the host’s circuit while detuning the tumor’s coherence [196].

#### Synthesis

Ultimately, the treatment of melanoma brain metastasis will depend on separating melanoma from the brain while preserving coexistence. All of melanoma’s adaptive mechanisms—vascular co-option, synaptic mimicry, metabolic coupling, and immune regulation—are maintained by continuous feedback from the neural environment. Therefore, therapy can disrupt melanoma’s equilibrium with the brain by removing these loops [22]. Therapeutic dissonance is the guiding principle here. It is the introduction of dissonance at the interfaces that allows reciprocal relationships between melanoma and the brain. This paradigm converts therapy from a confrontational model to a corrective one—a concerted effort to disconnect while preserving the structural integrity of the brain and disassembling the logic of connection. Thus, the defeat of intracranial melanoma will not derive from the intensity of cytotoxicity but from the precision of information and rhythm—learning the melody of the neural environment and altering only the musical notes that are no longer part of it [197].

## 8. Conclusions: The Quiet Architecture of Return

The concept of melanoma brain metastasis represents an evolutionary adaptation to specific areas of the central nervous system as opposed to a completely vascular or immune-related escape event. The evidence discussed above suggests that the neural crest developmental memory of melanomas provides a pre-existing chromatin environment for the re-expression of neuroectodermal gene expression programs, which in the brain are selectively supported by neurovascular and glial signals. As a result, at the blood–brain barrier/perivascular interface, melanoma cells are able to exploit integrins, chemokines, proteases, and other factors to migrate through the basement membrane and co-opt the earliest stages of vascular formation to establish a stable position in high-oxygen and nutrient availability microdomains. Upon establishment of a stable position within these microdomains, melanoma cells often express neurotransmitter receptors, ion channels, and axon guidance proteins, allowing for the activation of calcium-based signaling mechanisms, movement, and changes in phenotype based upon environmental stimuli. From a metabolic perspective, a significant number of lesions have been found to utilize lactate-supported oxidative phosphorylation to optimize their energy production capabilities in alignment with the oxidative capacity of the brain, while others continue to utilize glycolysis or exhibit hypoxic adaptations indicative of a highly variable metabolic repertoire in the brain. Additionally, the CNS has been shown to maintain its own form of immune tolerance towards melanomas through melanoma-specific checkpoints and through micro-glial programming. While the synaptic-adjacent molecules represent potential modulators of immune responses in melanomas, there is currently no in vivo data demonstrating the causal role of such molecules in regulating melanoma-specific immune tolerance. Therefore, the collective data supports a model of tumor–host interaction where molecular, electrical, metabolic, and neuro-immune interactions all contribute to the development and treatment resistance of melanoma brain metastasis.

While the proposed framework for understanding the convergence of various levels of biological organization is useful in organizing our current understanding of melanoma brain metastasis, it also leaves several important questions unanswered. First, the degree and duration of electrophysiological communication between neurons and melanoma cells in the brain has not been clearly established; whereas most evidence regarding the relationship between neuronal activity and melanoma cell function was obtained in vitro or in co-cultures of melanoma and neuronal cells, few studies have demonstrated a direct correlation between the two in vivo. Second, while a number of developmental and metabolic programs have been identified as being activated in melanoma cells that metastasize to the brain, the degree of heterogeneity among patients and among individual lesions suggests that these programs should be considered to be recurrent but not universal. Third, the relative contributions of perivascular signals, glial trophic programs, ion channel states, and melanoma-specific immune checkpoints are still not well defined and thus do not allow for the confident assignment of priorities among potential therapeutic targets.

Therefore, the next steps in defining the research agenda for melanoma brain metastasis include the following: (1) melanoma-specific in vivo experiments that utilize single-cell/spatial multi-omics in conjunction with either electrophysiology or calcium imaging to assess the dependence of melanoma cells on neuronal activity, ion channels, and synaptic-adjacent ligands; (2) spatially resolved metabolic mapping (metabolomic analysis and isotopic labeling) to stratify melanoma cells into those that utilize oxidative phosphorylation, glycolysis, and those that have a combination of both metabolic states and to correlate these states with vascular proximity, clonal lineage, and treatment history; (3) experimental approaches to understand how micro-glial and astrocytic programming influence melanoma-induced immune suppression using lineage-restricted models to distinguish between primary immune drivers and secondary, synaptic modulating effects; and (4) assessment of the efficacy of interface disruption therapies (adhesion/mechanotransduction inhibition, metabolic transfer interruption, and time-based regimens) as testable hypotheses for the treatment of melanoma brain metastasis, with the goal of developing a more accurate, evidence-based neuro-oncology approach to treating melanoma brain metastasis.

## Figures and Tables

**Table 1:** This table intends to outline the hierarchical structure of how melanoma reactivates the neural crest lineage program in the brain at the molecular, cellular, and network levels. At each scale, this research indicates a development continuum from reopen enhancers to integration into functional neural circuits, implying that metastatic melanoma is an activation of the embryonic plan as opposed to random adaptations.

Level of Organization	Key Features and Mechanisms	Physiological/Pathophysiological Context	Experimental or Clinical Correlates	Representative References
Epigenetic and transcriptional reactivation	Reopening of neural crest enhancers via H3K27ac deposition and SWI/SNF (BAF53B and ACTL6B) remodeling; cyclic SOX10–MITF–BRN2 triad maintains metastable melanocytic–neuroadaptive states	Reactivation of developmental chromatin landscapes under neural morphogen exposure (WNT7A, SHH, BDNF)	ATAC-seq, ChIP-seq, and scRNA-seq showing enhancer reactivation and lineage cycling in brain metastases	[53]
Transcriptional circuitry and non-coding regulation	Coordinated expression of proneural (ASCL1 and NEUROD1) and neural crest (SOX10, PAX3, FOXD3) genes; regulation by lncRNAs (SAMMSON and NEAT1) and miRNAs (miR-211 and miR-182/183/96)	Balances oxidative stress, migration, and neuroplastic signaling Within the brain microenvironment	Multi-omics and CRISPR perturbation studies revealing ncRNA–transcription factor coupling	[54]
Neuroadaptive proteomic convergence	De novo expression of synaptic proteins (NLGN3, CNTNAP2, SYN1, STXBP1) and vesicular fusion machinery (VAMP2 and RIM1)	Enables pseudo-synaptic coupling and neurotransmitter-mediated communication between melanoma and neurons	Proteomics and EM imaging demonstrating synaptic mimicry in intracranial metastases	[55]
Electrochemical and biomechanical signaling	ORAI1–STIM1 and TRPM7/TRPV4-mediated Ca^2+^ oscillations; integrin–laminin–FAK axis restores polarity and guides perivascular migration	Electric and mechanical feedback synchronizes melanoma clusters with local neuronal rhythms	Microelectrode array recordings and live-cell calcium imaging in co-culture systems	[56]
Systems-level integration and metastasis phenotype	Emergence of hybrid “neural–melanocytic” state capable of neurotransmitter release (glutamate and ATP) and metabolic coupling with glia	Establishes partially integrated neural subsystems that exploit neuronal circuitry for survival and spread	In vivo imaging and metabolic tracing of functional connectivity within metastases	[57]

**Table 2:** This table illustrates how melanoma has progressively adapted from initial structural adherence to electrophysiological assimilation in the brain’s electrical networks. The tumor adapts across five successive phases: (1) junction formation; (2) vesicle autonomy; (3) astrocyte maturation; (4) synchronized bioelectric activity; and (5) circuit-level plasticity. In doing so, the tumor progresses from being a foreign invader to becoming partially integrated in the neural network and utilizes the synaptically derived logic used by neurons for the purposes of malignant persistence.

Phase	Core Mechanisms and Molecular Features	Functional Integration Within Neural Microenvironment	Emergent Biological Consequences	Representative References
I. Junctional Genesis	Assembly of neurexin–neuroligin, LRRTM, and Slitrk complexes; selective heparan sulfate editing modulates adhesion specificity; bidirectional Eph–ephrin signaling organizes actin geometry and intercellular spacing	Establishes pseudo-synaptic interfaces that recapitulate excitatory and inhibitory synaptic architectures	Anchors melanoma cells to neuronal membranes and primes bidirectional neurotransmission	[114]
II. Vesicular Autonomy	SNARE-driven vesicle priming (syntaxin-1A, SNAP25, VAMP2) with Munc13/UNC13 scaffolding; PSD enrichment (DLG4, CaMKII, calcineurin); dual glutamatergic and GABAergic receptor sets	Enables semi-autonomous vesicle cycling and receptor-dependent responsiveness to neuronal activity	Melanoma gains capacity for chemical signaling and feedback modulation of local circuits	[115]
III. Astrocytic Maturation and Matrix Remodeling	Co-option of thrombospondin-1/2, hevin, and glypican 4/6; neuronal pentraxins increase receptor density; tumor-secreted metalloproteases reshape perisynaptic ECM (brevican and aggrecan)	Astrocyte-derived cues drive functional maturation of hybrid junctions and diffusion-regulated signal stability	Produces structurally competent and electrically responsive tumor–neuron synapses	[116]
IV. Bioelectric Coupling and Metabolic Synchrony	Connexin-43/30 gap junctions enable astrocytic cross-talk; ephaptic coupling mediates field transfer; Ca^2+^ dynamics via ORAI1–STIM1 and TRPM7/TRPV4; mitochondrial oscillations through OPA1–DRP1 feedback	Establishes electro-metabolic coherence, synchronizing ionic flux with mitochondrial ATP output	Enables sustained signaling and mesoscopic synchronization with neighboring neuronal populations	[117]
V. Circuit Plasticity and Information Exchange	Melanoma-mediated release of D-serine, glutamate, and ATP modulates dendritic thresholds; Hebbian-like plasticity (CaMKII–stargazin, calcineurin); TNT-mediated long-range coupling; LOX-driven mechanoelectrical tuning	Melanoma participates in dynamic circuit adaptation and feedback regulation of excitatory–inhibitory balance	Hybrid motifs (neuron–melanoma–astrocyte) sustain tumor survival while preserving circuit homeostasis	[118]

## Data Availability

The data presented in this study are available on request from the corresponding author.

## References

[1] Rice R., Cebra-Thomas J., Haugas M., Partanen J., Rice D.P.C., Gilbert S.F. (2017). Melanoblast Development Coincides with the Late Emerging Cells from the Dorsal Neural Tube in Turtle Trachemys Scripta. Sci. Rep..

[2] Tempone M.H., Borges-Martins V.P., César F., Alexandrino-Mattos D.P., de Figueiredo C.S., Raony Í., dos Santos A.A., Duarte-Silva A.T., Dias M.S., Freitas H.R. (2024). The Healthy and Diseased Retina Seen through Neuron–Glia Interactions. Int. J. Mol. Sci..

[3] Ceol C.J. (2023). Microenvironmental GABA Signaling Regulates Melanomagenesis through Reciprocal Melanoma–Keratinocyte Communication. Cancer Discov..

[4] Végh A.G., Fazakas C., Nagy K., Wilhelm I., Molnár J., Krizbai I.A., Szegletes Z., Váró G. (2012). Adhesion and Stress Relaxation Forces between Melanoma and Cerebral Endothelial Cells. Eur. Biophys. J. EBJ.

[5] Zhang P., Goodrich C., Fu C., Dong C. (2014). Melanoma Upregulates ICAM-1 Expression on Endothelial Cells through Engagement of Tumor CD44 with Endothelial E-Selectin and Activation of a PKCα–P38–SP-1 Pathway. FASEB J..

[6] Ruan J., McKee K.K., Yurchenco P.D., Yao Y. (2022). Exogenous Laminin Exhibits a Unique Vascular Pattern in the Brain via Binding to Dystroglycan and Integrins. Fluids Barriers CNS.

[7] Stejerean-Todoran I., Zimmermann K., Gibhardt C.S., Vultur A., Ickes C., Shannan B., Bonilla del Rio Z., Wölling A., Cappello S., Sung H. (2022). MCU Controls Melanoma Progression through a Redox-controlled Phenotype Switch. EMBO Rep..

[8] Balinda H.U., Sedgwick A., D’Souza-Schorey C. (2022). Mechanisms Underlying Melanoma Invasion as a Consequence of MLK3 Loss. Exp. Cell Res..

[9] Nickerson K.R., Sammoura F.M., Zhou Y., Jaworski A. (2025). Slit-Robo Signaling Supports Motor Neuron Avoidance of the Spinal Cord Midline through DCC Antagonism and Other Mechanisms. Front. Cell Dev. Biol..

[10] Caruso G., Garcia Moreira C.G., Iaboni E., Tripodo M., Ferrarotto R., Abbritti R.V., Conte L., Caffo M. (2025). Tumor Microenvironment in Melanoma Brain Metastasis: A New Potential Target?. Int. J. Mol. Sci..

[11] Pu T., Sun J., Ren G., Li H. (2025). Neuro-Immune Crosstalk in Cancer: Mechanisms and Therapeutic Implications. Signal Transduct. Target. Ther..

[12] Spurling D., Anchan A., Hucklesby J., Finlay G., Angel C.E., Graham E.S. (2023). Melanoma Cells Produce Large Vesicular-Bodies That Cause Rapid Disruption of Brain Endothelial Barrier-Integrity and Disassembly of Junctional Proteins. Int. J. Mol. Sci..

[13] Di Maio V., Ventriglia F., Santillo S. (2016). A Model of Cooperative Effect of AMPA and NMDA Receptors in Glutamatergic Synapses. Cogn. Neurodyn..

[14] Ramachandran R., Jeans A.F. (2025). Breaking Down Glioma-Microenvironment Crosstalk. Neuroscientist.

[15] Marrapodi R., Kovacs D., Migliano E., Caputo S., Papaccio F., Pallara T., Cota C., Bellei B. (2025). Melanoma–Keratinocyte Crosstalk Participates in Melanoma Progression with Mechanisms Partially Overlapping with Those of Cancer-Associated Fibroblasts. Int. J. Mol. Sci..

[16] Zhou X., Yan T., Huang C., Xu Z., Wang L., Jiang E., Wang H., Chen Y., Liu K., Shao Z. (2018). Melanoma Cell-Secreted Exosomal miR-155-5p Induce Proangiogenic Switch of Cancer-Associated Fibroblasts via SOCS1/JAK2/STAT3 Signaling Pathway. J. Exp. Clin. Cancer Res..

[17] Strackeljan L., Baczynska E., Cangalaya C., Baidoe-Ansah D., Wlodarczyk J., Kaushik R., Dityatev A. (2021). Microglia Depletion-Induced Remodeling of Extracellular Matrix and Excitatory Synapses in the Hippocampus of Adult Mice. Cells.

[18] Biermann J., Melms J.C., Amin A.D., Wang Y., Caprio L.A., Karz A., Tagore S., Barrera I., Ibarra-Arellano M.A., Andreatta M. (2022). Dissecting the Treatment-Naïve Ecosystem of Human Melanoma Brain Metastasis. Cell.

[19] Radke J., Schumann E., Onken J., Koll R., Acker G., Bodnar B., Senger C., Tierling S., Möbs M., Vajkoczy P. (2022). Decoding Molecular Programs in Melanoma Brain Metastases. Nat. Commun..

[20] Fager A., Samuelsson M., Olofsson Bagge R., Pivodic A., Bjursten S., Levin M., Jespersen H., Ny L. (2025). Immune Checkpoint Inhibitor Therapy Is Associated with a Decreased Risk of Developing Melanoma Brain Metastases. BJC Rep..

[21] Gorodetska I., Schulz A., Behre G., Dubrovska A. (2025). Confronting Melanoma Radioresistance: Mechanisms and Therapeutic Strategies. Cancers.

[22] Bonzano E., Barruscotti S., Chiellino S., Montagna B., Bonzano C., Imarisio I., Colombo S., Guerrini F., Saddi J., La Mattina S. (2025). Current Treatment Paradigms for Advanced Melanoma with Brain Metastases. Int. J. Mol. Sci..

[23] Li Y., Liu F., Cai Q., Deng L., Ouyang Q., Zhang X.H.-F., Zheng J. (2025). Invasion and Metastasis in Cancer: Molecular Insights and Therapeutic Targets. Signal Transduct. Target. Ther..

[24] Levin M. (2019). The Computational Boundary of a “Self”: Developmental Bioelectricity Drives Multicellularity and Scale-Free Cognition. Front. Psychol..

[25] Sabău A.-H., Tinca A.-C., Niculescu R., Cocuz I.G., Cozac-Szöke A.R., Lazar B.A., Chiorean D.M., Budin C.E., Cotoi O.S. (2025). Cancer Stem Cells in Melanoma: Drivers of Tumor Plasticity and Emerging Therapeutic Strategies. Int. J. Mol. Sci..

[26] Méndez-Maldonado K., Vega-López G.A., Aybar M.J., Velasco I. (2020). Neurogenesis from Neural Crest Cells: Molecular Mechanisms in the Formation of Cranial Nerves and Ganglia. Front. Cell Dev. Biol..

[27] Knyazeva A., Dyachuk V. (2024). Neural Crest and Sons: Role of Neural Crest Cells and Schwann Cell Precursors in Development and Gland Embryogenesis. Front. Cell Dev. Biol..

[28] Baloiu A.I., Filipoiu F., Toader C., Covache-Busuioc R.-A., Munteanu O., Serban M. (2025). Sphenoid Sinus Hyperpneumatization: Anatomical Variants, Molecular Blueprints, and AI-Augmented Roadmaps for Skull Base Surgery. Front. Endocrinol..

[29] Green K.J., Niessen C.M., Rübsam M., Perez White B.E., Broussard J.A. (2022). The Desmosome-Keratin Scaffold Integrates ErbB Family and Mechanical Signaling to Polarize Epidermal Structure and Function. Front. Cell Dev. Biol..

[30] Hon G.C., Rajagopal N., Shen Y., McCleary D.F., Yue F., Dang M.D., Ren B. (2013). Adult Tissue Methylomes Harbor Epigenetic Memory at Embryonic Enhancers. Nat. Genet..

[31] Spinella F., Caprara V., Cianfrocca R., Rosanò L., Di Castro V., Garrafa E., Natali P.G., Bagnato A. (2014). The Interplay between Hypoxia, Endothelial and Melanoma Cells Regulates Vascularization and Cell Motility through Endothelin-1 and Vascular Endothelial Growth Factor. Carcinogenesis.

[32] Avitabile M., Succoio M., Testori A., Cardinale A., Vaksman Z., Lasorsa V.A., Cantalupo S., Esposito M., Cimmino F., Montella A. (2020). Neural Crest-Derived Tumor Neuroblastoma and Melanoma Share 1p13.2 as Susceptibility Locus That Shows a Long-Range Interaction with the SLC16A1 Gene. Carcinogenesis.

[33] Nygaard V., Prasmickaite L., Vasiliauskaite K., Clancy T., Hovig E. (2014). Melanoma Brain Colonization Involves the Emergence of a Brain-Adaptive Phenotype. Oncoscience.

[34] Rembiałkowska N., Rekiel K., Urbanowicz P., Mamala M., Marczuk K., Wojtaszek M., Żywica M., Radzevičiūtė-Valčiukė E., Novickij V., Kulbacka J. (2025). Epigenetic Dysregulation in Cancer: Implications for Gene Expression and DNA Repair-Associated Pathways. Int. J. Mol. Sci..

[35] Kennedy L.B., Van Swearingen A.E.D., Lee M.R., Rogers L.W., Sibley A.B., Sheng J., Zhang D., Qin X., Lipp E.S., Kumar S. (2025). A Comprehensive, Multi-Center, Immunogenomic Analysis of Melanoma Brain Metastases. Acta Neuropathol. Commun..

[36] Jäger K., Larribère L., Wu H., Weiss C., Gebhardt C., Utikal J. (2019). Expression of Neural Crest Markers GLDC and ERRFI1 Is Correlated with Melanoma Prognosis. Cancers.

[37] McConnell A.M., Mito J.K., Ablain J., Dang M., Formichella L., Fisher D.E., Zon L.I. (2019). Neural Crest State Activation in NRAS Driven Melanoma, but Not in NRAS-Driven Melanocyte Expansion. Dev. Biol..

[38] Voicu V., Toader C., Șerban M., Covache-Busuioc R.-A., Ciurea A.V. (2025). Systemic Neurodegeneration and Brain Aging: Multi-Omics Disintegration, Proteostatic Collapse, and Network Failure Across the CNS. Biomedicines.

[39] Lai X., Luan C., Zhang Z., Wessely A., Heppt M.V., Berking C., Vera J. (2025). SOX10, MITF, and microRNAs: Decoding Their Interplay in Regulating Melanoma Plasticity. Int. J. Cancer.

[40] Kunoh S., Nakashima H., Nakashima K. (2024). Epigenetic Regulation of Neural Stem Cells in Developmental and Adult Stages. Epigenomes.

[41] Zeinelabdeen Y., Abaza T., Yasser M.B., Elemam N.M., Youness R.A. (2024). MIAT LncRNA: A Multifunctional Key Player in Non-Oncological Pathological Conditions. Non-Coding RNA Res..

[42] DiNapoli S.E., Martinez-McFaline R., Shen H., Doane A.S., Perez A.R., Verma A., Simon A., Nelson I., Balgobin C.A., Bourque C.T. (2022). Histone 3 Methyltransferases Alter Melanoma Initiation and Progression Through Discrete Mechanisms. Front. Cell Dev. Biol..

[43] Alammari F., Al-Hujaily E.M., Alshareeda A., Albarakati N., Al-Sowayan B.S. (2024). Hidden Regulators: The Emerging Roles of lncRNAs in Brain Development and Disease. Front. Neurosci..

[44] Váraljai R., Horn S., Sucker A., Piercianek D., Schmitt V., Carpinteiro A., Becker K.A., Reifenberger J., Roesch A., Felsberg J. (2021). Integrative Genomic Analyses of Patient-Matched Intracranial and Extracranial Metastases Reveal a Novel Brain-Specific Landscape of Genetic Variants in Driver Genes of Malignant Melanoma. Cancers.

[45] Toader C., Serban M., Munteanu O., Covache-Busuioc R.-A., Enyedi M., Ciurea A.V., Tataru C.P. (2025). From Synaptic Plasticity to Neurodegeneration: BDNF as a Transformative Target in Medicine. Int. J. Mol. Sci..

[46] Xie Y., Kuan A.T., Wang W., Herbert Z.T., Mosto O., Olukoya O., Adam M., Vu S., Kim M., Tran D. (2022). Astrocyte-Neuron Crosstalk through Hedgehog Signaling Mediates Cortical Synapse Development. Cell Rep..

[47] Tagliaferro M., Ponti D. (2023). The Signaling of Neuregulin-Epidermal Growth Factor Receptors and Its Impact on the Nervous System. Neuroglia.

[48] Maneshi M.M., Toth A.B., Ishii T., Hori K., Tsujikawa S., Shum A.K., Shrestha N., Yamashita M., Miller R.J., Radulovic J. (2020). Orai1 Channels Are Essential for Amplification of Glutamate-Evoked Ca^2+^ Signals in Dendritic Spines to Regulate Working and Associative Memory. Cell Rep..

[49] Trujillo C.A., Gao R., Negraes P.D., Gu J., Buchanan J., Preissl S., Wang A., Wu W., Haddad G.G., Chaim I.A. (2019). Complex Oscillatory Waves Emerging from Cortical Organoids Model Early Human Brain Network Development. Cell Stem Cell.

[50] Liu Q.-Q., Dong Z.-K., Wang Y.-F., Jin W.-L. (2025). Reprogramming Neural-Tumor Crosstalk: Emerging Therapeutic Dimensions and Targeting Strategies. Mil. Med. Res..

[51] Pinto M., Violante S., Cascão R., Faria C.C. (2025). Unlocking the Role of Metabolic Pathways in Brain Metastatic Disease. Cells.

[52] Kulesa P.M., Morrison J.A., Bailey C.M. (2013). The Neural Crest and Cancer: A Developmental Spin on Melanoma. Cells Tissues Organs.

[53] Zhao W., Xu Y., Wang Y., Gao D., King J., Xu Y., Liang F.-S. (2021). Investigating Crosstalk between H3K27 Acetylation and H3K4 Trimethylation in CRISPR/dCas-Based Epigenome Editing and Gene Activation. Sci. Rep..

[54] Șerban M., Toader C., Covache-Busuioc R.-A. (2025). CRISPR and Artificial Intelligence in Neuroregeneration: Closed-Loop Strategies for Precision Medicine, Spinal Cord Repair, and Adaptive Neuro-Oncology. Int. J. Mol. Sci..

[55] Pronot M., Kieffer F., Gay A.-S., Debayle D., Forquet R., Poupon G., Schorova L., Martin S., Gwizdek C. (2021). Proteomic Identification of an Endogenous Synaptic SUMOylome in the Developing Rat Brain. Front. Mol. Neurosci..

[56] Sun J., Lu F., He H., Shen J., Messina J., Mathew R., Wang D., Sarnaik A.A., Chang W.-C., Kim M. (2014). STIM1- and Orai1-Mediated Ca^2+^ Oscillation Orchestrates Invadopodium Formation and Melanoma Invasion. J. Cell Biol..

[57] McGrath T., Baskerville R., Rogero M., Castell L. (2022). Emerging Evidence for the Widespread Role of Glutamatergic Dysfunction in Neuropsychiatric Diseases. Nutrients.

[58] Saltarin F., Wegmüller A., Bejarano L., Ildiz E.S., Zwicky P., Vianin A., Spadin F., Soukup K., Wischnewski V., Engelhardt B. (2023). Compromised Blood-Brain Barrier Junctions Enhance Melanoma Cell Intercalation and Extravasation. Cancers.

[59] Izraely S., Ben-Menachem S., Sagi-Assif O., Meshel T., Malka S., Telerman A., Bustos M.A., Ramos R.I., Pasmanik-Chor M., Hoon D.S.B. (2021). The Melanoma Brain Metastatic Microenvironment: Aldolase C Partakes in Shaping the Malignant Phenotype of Melanoma Cells—A Case of Inter-tumor Heterogeneity. Mol. Oncol..

[60] Toader C., Dumitru A.V., Eva L., Serban M., Covache-Busuioc R.-A., Ciurea A.V. (2024). Nanoparticle Strategies for Treating CNS Disorders: A Comprehensive Review of Drug Delivery and Theranostic Applications. Int. J. Mol. Sci..

[61] Adamiak-Nikolouzou K., Słomiński A.T., Skalska Z., Inkielewicz-Stępniak I. (2025). Therapeutic Use of Integrin Signaling in Melanoma Cells: Physical Link with the Extracellular Matrix (ECM). Cancers.

[62] Wu Z., Bian Y., Chu T., Wang Y., Man S., Song Y., Wang Z. (2022). The Role of Angiogenesis in Melanoma: Clinical Treatments and Future Expectations. Front. Pharmacol..

[63] Kanai R., Norton E., Stern P., Hynes R.O., Lamar J.M. (2024). Identification of a Gene Signature That Predicts Dependence upon YAP/TAZ-TEAD. Cancers.

[64] Barkovskaya A., Buffone A., Žídek M., Weaver V.M. (2020). Proteoglycans as Mediators of Cancer Tissue Mechanics. Front. Cell Dev. Biol..

[65] Anfuso C.D., Longo A., Distefano A., Amorini A.M., Salmeri M., Zanghì G., Giallongo C., Giurdanella G., Lupo G. (2020). Uveal Melanoma Cells Elicit Retinal Pericyte Phenotypical and Biochemical Changes in an in Vitro Model of Coculture. Int. J. Mol. Sci..

[66] Nadhan R., Nath K., Basu S., Isidoro C., Song Y.S., Dhanasekaran D.N. (2025). Decoding Lysophosphatidic Acid Signaling in Physiology and Disease: Mapping the Multimodal and Multinodal Signaling Networks. Signal Transduct. Target. Ther..

[67] Niveau C., Sosa Cuevas E., Saas P., Aspord C. (2024). Glycans in Melanoma: Drivers of Tumour Progression but Sweet Targets to Exploit for Immunotherapy. Immunology.

[68] Brás M.M., Radmacher M., Sousa S.R., Granja P.L. (2020). Melanoma in the Eyes of Mechanobiology. Front. Cell Dev. Biol..

[69] Șerban M., Toader C., Covache-Busuioc R.-A. (2025). The Collapse of Brain Clearance: Glymphatic-Venous Failure, Aquaporin-4 Breakdown, and AI-Empowered Precision Neurotherapeutics in Intracranial Hypertension. Int. J. Mol. Sci..

[70] Hashemi G., Dight J., Khosrotehrani K., Sormani L. (2022). Melanoma Tumour Vascularization and Tissue-Resident Endothelial Progenitor Cells. Cancers.

[71] Toader C., Serban M., Covache-Busuioc R.-A., Radoi M.P., Aljboor G.S.R., Costin H.P., Corlatescu A.D., Glavan L.-A., Gorgan R.M. (2024). Cerebellar Cavernoma Resection: Case Report with Long-Term Follow-Up. J. Clin. Med..

[72] Yan W., Wang Y., Chen Y., Guo Y., Li Q., Wei X. (2021). Exosomal miR-130b-3p Promotes Progression and Tubular Formation Through Targeting PTEN in Oral Squamous Cell Carcinoma. Front. Cell Dev. Biol..

[73] Abdelsalam M., Ahmed M., Osaid Z., Hamoudi R., Harati R. (2023). Insights into Exosome Transport through the Blood–Brain Barrier and the Potential Therapeutical Applications in Brain Diseases. Pharmaceuticals.

[74] Fernández-Cortés M., Delgado-Bellido D., Bermúdez-Jiménez E., Paramio J.M., O’Valle F., Vinckier S., Carmeliet P., Garcia-Diaz A., Oliver F.J. (2023). PARP Inhibition Promotes Endothelial-like Traits in Melanoma Cells and Modulates Pericyte Coverage Dynamics during Vasculogenic Mimicry. J. Pathol..

[75] Indini A., Grossi F., Mandalà M., Taverna D., Audrito V. (2021). Metabolic Interplay between the Immune System and Melanoma Cells: Therapeutic Implications. Biomedicines.

[76] Untiveros G., Raskind A., Linares L., Dotti A., Strizzi L. (2022). Netrin-1 Stimulates Migration of Neogenin Expressing Aggressive Melanoma Cells. Int. J. Mol. Sci..

[77] Luty M., Szydlak R., Pabijan J., Zemła J., Oevreeide I.H., Prot V.E., Stokke B.T., Lekka M., Zapotoczny B. (2024). Tubulin-Targeted Therapy in Melanoma Increases the Cell Migration Potential by Activation of the Actomyosin Cytoskeleton─An In Vitro Study. ACS Biomater. Sci. Eng..

[78] Krummel D.A.P., Nasti T.H., Kaluzova M., Kallay L., Bhattacharya D., Melms J.C., Izar B., Xu M., Burnham A., Ahmed T. (2021). Melanoma Cell Intrinsic GABAA Receptor Enhancement Potentiates Radiation and Immune Checkpoint Inhibitor Response by Promoting Direct and T Cell-Mediated Anti-Tumor Activity. Int. J. Radiat. Oncol. Biol. Phys..

[79] Șerban M., Toader C., Covache-Busuioc R.-A. (2025). Blueprint of Collapse: Precision Biomarkers, Molecular Cascades, and the Engineered Decline of Fast-Progressing ALS. Int. J. Mol. Sci..

[80] Keskinov A.A., Tapias V., Watkins S.C., Ma Y., Shurin M.R., Shurin G.V. (2016). Impact of the Sensory Neurons on Melanoma Growth In Vivo. PLoS ONE.

[81] Paunikar S., Tamagnone L. (2024). Connexin-43 in Cancer: Above and Beyond Gap Junctions!. Cancers.

[82] Șerban M., Toader C., Covache-Busuioc R.-A. (2025). Anatomy-Guided Microsurgical Resection of a Dominant Frontal Lobe Tumor Without Intraoperative Adjuncts: A Case Report from a Resource-Limited Context. Diagnostics.

[83] Farah C., Mignion L., Jordan B.F. (2024). Metabolic Profiling to Assess Response to Targeted and Immune Therapy in Melanoma. Int. J. Mol. Sci..

[84] Nowacka A., Fajkiel-Madajczyk A., Ohla J., Woźniak-Dąbrowska K., Liss S., Gryczka K., Smuczyński W., Ziółkowska E., Bożiłow D., Śniegocki M. (2023). Current Treatment of Melanoma Brain Metastases. Cancers.

[85] Toader C., Tataru C.P., Munteanu O., Covache-Busuioc R.-A., Serban M., Ciurea A.V., Enyedi M. (2024). Revolutionizing Neuroimmunology: Unraveling Immune Dynamics and Therapeutic Innovations in CNS Disorders. Int. J. Mol. Sci..

[86] Tenchov R., Sasso J.M., Wang X., Zhou Q.A. (2024). Aging Hallmarks and Progression and Age-Related Diseases: A Landscape View of Research Advancement. ACS Chem. Neurosci..

[87] Abate-Daga D., Ramello M.C., Smalley I., Forsyth P.A., Smalley K.S.M. (2018). The Biology and Therapeutic Management of Melanoma Brain Metastases. Biochem. Pharmacol..

[88] Buga A.-M., Docea A.O., Albu C., Malin R.D., Branisteanu D.E., Ianosi G., Ianosi S.L., Iordache A., Calina D. (2019). Molecular and Cellular Stratagem of Brain Metastases Associated with Melanoma. Oncol. Lett..

[89] Tuomela K., Ambrose A.R., Davis D.M. (2022). Escaping Death: How Cancer Cells and Infected Cells Resist Cell-Mediated Cytotoxicity. Front. Immunol..

[90] Zhang Y., Liao Q., Wen X., Fan J., Yuan T., Tong X., Jia R., Chai P., Fan X. (2025). Hijacking of the Nervous System in Cancer: Mechanism and Therapeutic Targets. Mol. Cancer.

[91] Farrugia B.L., Melrose J. (2023). The Glycosaminoglycan Side Chains and Modular Core Proteins of Heparan Sulphate Proteoglycans and the Varied Ways They Provide Tissue Protection by Regulating Physiological Processes and Cellular Behaviour. Int. J. Mol. Sci..

[92] Toader C., Serban M., Covache-Busuioc R.-A., Radoi M.P., Aljboor G.S.R., Costin H.P., Ilie M.-M., Popa A.A., Gorgan R.M. (2025). Single-Stage Microsurgical Clipping of Multiple Intracranial Aneurysms in a Patient with Cerebral Atherosclerosis: A Case Report and Review of Surgical Management. J. Clin. Med..

[93] Maidorn M., Olichon A., Rizzoli S.O., Opazo F. (2019). Nanobodies Reveal an Extra-Synaptic Population of SNAP-25 and Syntaxin 1A in Hippocampal Neurons. mAbs.

[94] Costas-Insua C., Seijo-Vila M., Blázquez C., Blasco-Benito S., Rodríguez-Baena F.J., Marsicano G., Pérez-Gómez E., Sánchez C., Sánchez-Laorden B., Guzmán M. (2023). Neuronal Cannabinoid CB1 Receptors Suppress the Growth of Melanoma Brain Metastases by Inhibiting Glutamatergic Signalling. Cancers.

[95] von Roemeling C.A., Radisky D.C., Marlow L.A., Cooper S.J., Grebe S.K., Anastasiadis P.Z., Tun H.W., Copland J.A. (2014). Neuronal Pentraxin 2 Supports Clear Cell Renal Cell Carcinoma by Activating the AMPA-Selective Glutamate Receptor-4. Cancer Res..

[96] Toader C., Brehar F.-M., Radoi M.P., Serban M., Covache-Busuioc R.-A., Glavan L.-A., Ciurea A.V., Dobrin N. (2024). The Microsurgical Resection of an Arteriovenous Malformation in a Patient with Thrombophilia: A Case Report and Literature Review. Diagnostics.

[97] Alqabandi J.A., David R., Abdel-Motal U.M., ElAbd R.O., Youcef-Toumi K. (2024). An Innovative Cellular Medicine Approach via the Utilization of Novel Nanotechnology-Based Biomechatronic Platforms as a Label-Free Biomarker for Early Melanoma Diagnosis. Sci. Rep..

[98] Sinha M., Narayanan R. (2015). HCN Channels Enhance Spike Phase Coherence and Regulate the Phase of Spikes and LFPs in the Theta-Frequency Range. Proc. Natl. Acad. Sci. USA.

[99] Orellana J.A., Froger N., Ezan P., Jiang J.X., Bennett M.V.L., Naus C.C., Giaume C., Sáez J.C. (2011). ATP and Glutamate Released via Astroglial Connexin 43 Hemichannels Mediate Neuronal Death through Activation of Pannexin 1 Hemichannels. J. Neurochem..

[100] Bockstiegel J., Engelhardt J., Weindl G. (2023). P2X7 Receptor Activation Leads to NLRP3-Independent IL-1β Release by Human Macrophages. Cell Commun. Signal..

[101] Bychkov M.L., Kirichenko A.V., Mikhaylova I.N., Paramonov A.S., Kirpichnikov M.P., Shulepko M.A., Lyukmanova E.N. (2022). Extracellular Vesicles Derived from Metastatic Melanoma Cells Transfer A7-nAChR mRNA, Thus Increasing the Surface Expression of the Receptor and Stimulating the Growth of Normal Keratinocytes. Acta Nat..

[102] Qiang Y., Artoni P., Seo K.J., Culaclii S., Hogan V., Zhao X., Zhong Y., Han X., Wang P.-M., Lo Y.-K. (2018). Transparent Arrays of Bilayer-Nanomesh Microelectrodes for Simultaneous Electrophysiology and Two-Photon Imaging in the Brain. Sci. Adv..

[103] Toader C., Serban M., Covache-Busuioc R.-A., Radoi M.P., Ciurea A.V., Dobrin N. (2025). Comprehensive Management of a Giant Left Frontal AVM Coexisting with a Bilobed PComA Aneurysm: A Case Report Highlighting Multidisciplinary Strategies and Advanced Neurosurgical Techniques. J. Clin. Med..

[104] Abbasian V., Davoudi S., Vahabzadeh A., Maftoon-Azad M.J., Janahmadi M. (2025). Astroglial Kir4.1 and AQP4 Channels: Key Regulators of Potassium Homeostasis and Their Implications in Autism Spectrum Disorders. Cell. Mol. Neurobiol..

[105] Abreu D.S., Gomes J.I., Ribeiro F.F., Diógenes M.J., Sebastião A.M., Vaz S.H. (2023). Astrocytes Control Hippocampal Synaptic Plasticity through the Vesicular-Dependent Release of D-Serine. Front. Cell. Neurosci..

[106] Șerban M., Toader C., Covache-Busuioc R.-A. (2025). Ruptured Posterior Inferior Cerebellar Artery Aneurysms: Integrating Microsurgical Expertise, Endovascular Challenges, and AI-Driven Risk Assessment. J. Clin. Med..

[107] Nagy-Pál P., Veres J.M., Fekete Z., Karlócai M.R., Weisz F., Barabás B., Reéb Z., Hájos N. (2023). Structural Organization of Perisomatic Inhibition in the Mouse Medial Prefrontal Cortex. J. Neurosci..

[108] Reyes-García S.E., Escobar M.L. (2021). Calcineurin Participation in Hebbian and Homeostatic Plasticity Associated with Extinction. Front. Cell. Neurosci..

[109] Ding Z., Ogata D., Roszik J., Qin Y., Kim S.-H., Tetzlaff M.T., Lazar A.J., Davies M.A., Ekmekcioglu S., Grimm E.A. (2021). iNOS Associates with Poor Survival in Melanoma: A Role for Nitric Oxide in the PI3K-AKT Pathway Stimulation and PTEN S-Nitrosylation. Front. Oncol..

[110] Guan F., Wu X., Zhou J., Lin Y., He Y., Fan C., Zeng Z., Xiong W. (2024). Mitochondrial Transfer in Tunneling Nanotubes—A New Target for Cancer Therapy. J. Exp. Clin. Cancer Res..

[111] Mierke C.T. (2024). Extracellular Matrix Cues Regulate Mechanosensing and Mechanotransduction of Cancer Cells. Cells.

[112] Andoh M., Shinoda N., Taira Y., Araki T., Kasahara Y., Takeuchi H., Miura M., Ikegaya Y., Koyama R. (2025). Nonapoptotic Caspase-3 Guides C1q-Dependent Synaptic Phagocytosis by Microglia. Nat. Commun..

[113] Sun W., Liu Z., Jiang X., Chen M.B., Dong H., Liu J., Südhof T.C., Quake S.R. (2024). Spatial Transcriptomics Reveal Neuron-Astrocyte Synergy in Long-Term Memory. Nature.

[114] D’Autréaux F., Chalazonitis A., Arumugam D., Gershon T., Gershon M.D. (2025). Contribution of Neuroligin and Neurexin Alternative Splicing to the Establishment of Enteric Neuronal Synaptic Specificity. Am. J. Physiol. Gastrointest. Liver Physiol..

[115] Kalyana Sundaram R.V., Jin H., Li F., Shu T., Coleman J., Yang J., Pincet F., Zhang Y., Rothman J.E., Krishnakumar S.S. (2021). Munc13 Binds and Recruits SNAP25 to Chaperone SNARE Complex Assembly. FEBS Lett..

[116] Zheng H., Feng Y., Tang J., Yu F., Wang Z., Xu J., Hai C., Jiang M., Cheng Y., Shao Z. (2025). Astrocyte-Secreted Cues Promote Neural Maturation and Augment Activity in Human Forebrain Organoids. Nat. Commun..

[117] Motori E., Atanassov I., Kochan S.M.V., Folz-Donahue K., Sakthivelu V., Giavalisco P., Toni N., Puyal J., Larsson N.-G. (2020). Neuronal Metabolic Rewiring Promotes Resilience to Neurodegeneration Caused by Mitochondrial Dysfunction. Sci. Adv..

[118] Bodner O., Radzishevsky I., Foltyn V.N., Touitou A., Valenta A.C., Rangel I.F., Panizzutti R., Kennedy R.T., Billard J.M., Wolosker H. (2020). D-Serine Signaling and NMDAR-Mediated Synaptic Plasticity Are Regulated by System A-Type of Glutamine/D-Serine Dual Transporters. J. Neurosci. Off. J. Soc. Neurosci..

[119] Tan L.Y., Cockshell M.P., Moore E., Myo Min K.K., Ortiz M., Johan M.Z., Ebert B., Ruszkiewicz A., Brown M.P., Ebert L.M. (2022). Vasculogenic Mimicry Structures in Melanoma Support the Recruitment of Monocytes. Oncoimmunology.

[120] Gkintoni E., Vassilopoulos S.P., Nikolaou G. (2025). Brain-Inspired Multisensory Learning: A Systematic Review of Neuroplasticity and Cognitive Outcomes in Adult Multicultural and Second Language Acquisition. Biomimetics.

[121] Tan I.J., Parikh A.K., Cohen B.A. (2024). Melanoma Metabolism: Molecular Mechanisms and Therapeutic Implications in Cutaneous Oncology. Cancer Med..

[122] Pouyan A., Ghorbanlo M., Eslami M., Jahanshahi M., Ziaei E., Salami A., Mokhtari K., Shahpasand K., Farahani N., Meybodi T.E. (2025). Glioblastoma Multiforme: Insights into Pathogenesis, Key Signaling Pathways, and Therapeutic Strategies. Mol. Cancer.

[123] Toader C., Brehar F.M., Radoi M.P., Serban M., Covache-Busuioc R.-A., Aljboor G.S., Gorgan R.M. (2024). The Management of a Giant Convexity En Plaque Anaplastic Meningioma with Gerstmann Syndrome: A Case Report of Surgical Outcomes in a 76-Year-Old Male. Diagnostics.

[124] Mancini S.J.C., Balabanian K., Corre I., Gavard J., Lazennec G., Le Bousse-Kerdilès M.-C., Louache F., Maguer-Satta V., Mazure N.M., Mechta-Grigoriou F. (2021). Deciphering Tumor Niches: Lessons from Solid and Hematological Malignancies. Front. Immunol..

[125] Șerban M., Toader C., Covache-Busuioc R.-A. (2025). Precision Neuro-Oncology in Glioblastoma: AI-Guided CRISPR Editing and Real-Time Multi-Omics for Genomic Brain Surgery. Int. J. Mol. Sci..

[126] Tang S., Yang B. (2025). The Diverse Roles of Mitochondria in Regulating Cancer Metastasis. Curr. Issues Mol. Biol..

[127] In G.K., Ribeiro J.R., Yin J., Xiu J., Bustos M.A., Ito F., Chow F., Zada G., Hwang L., Salama A.K.S. (2023). Multi-Omic Profiling Reveals Discrepant Immunogenic Properties and a Unique Tumor Microenvironment among Melanoma Brain Metastases. Npj Precis. Oncol..

[128] Colby L., Preskitt C., Ho J.S., Balsara K., Wu D. (2025). Brain Metastasis: A Literary Review of the Possible Relationship Between Hypoxia and Angiogenesis in the Growth of Metastatic Brain Tumors. Int. J. Mol. Sci..

[129] Barba I., Carrillo-Bosch L., Seoane J. (2024). Targeting the Warburg Effect in Cancer: Where Do We Stand?. Int. J. Mol. Sci..

[130] Du F., Yang L., Liu J., Wang J., Fan L., Duangmano S., Liu H., Liu M., Wang J., Zhong X. (2023). The Role of Mitochondria in the Resistance of Melanoma to PD-1 Inhibitors. J. Transl. Med..

[131] Toader C., Brehar F.-M., Radoi M.P., Serban M., Covache-Busuioc R.-A., Aljboor G.S., Gorgan R.M. (2025). Stroke and Pulmonary Thromboembolism Complicating a Kissing Aneurysm in the M1 Segment of the Right MCA. J. Clin. Med..

[132] Sassano M.L., Felipe-Abrio B., Agostinis P. (2022). ER-Mitochondria Contact Sites; a Multifaceted Factory for Ca^2+^ Signaling and Lipid Transport. Front. Cell Dev. Biol..

[133] Giannitti G., Paganoni A.J.J., Marchesi S., Garavaglia R., Fontana F. (2025). Mitochondrial Bioenergetics and Networks in Melanoma: An Update. Apoptosis.

[134] Toader C., Tataru C.P., Munteanu O., Serban M., Covache-Busuioc R.-A., Ciurea A.V., Enyedi M. (2024). Decoding Neurodegeneration: A Review of Molecular Mechanisms and Therapeutic Advances in Alzheimer’s, Parkinson’s, and ALS. Int. J. Mol. Sci..

[135] Chang L., Qin C., Wu J., Jiang H., Xu Q., Chen J., Xu X., Zhang X., Guan M., Deng X. (2025). The Crosstalk between Glutathione Metabolism and Non-Coding RNAs in Cancer Progression and Treatment Resistance. Redox Biol..

[136] Șerban M., Toader C., Covache-Busuioc R.-A. (2025). The Redox Revolution in Brain Medicine: Targeting Oxidative Stress with AI, Multi-Omics and Mitochondrial Therapies for the Precision Eradication of Neurodegeneration. Int. J. Mol. Sci..

[137] Diaconescu I.B., Dumitru A.V., Tataru C.P., Toader C., Șerban M., Covache-Busuioc R.-A., Eva L. (2025). From Electron Imbalance to Network Collapse: Decoding the Redox Code of Ischemic Stroke for Biomarker-Guided Precision Neuroprotection. Int. J. Mol. Sci..

[138] Apryatin S.A. (2025). The Neurometabolic Function of the Dopamine–Aminotransferase System. Metabolites.

[139] Tagore M., Hergenreder E., Perlee S.C., Cruz N.M., Menocal L., Suresh S., Chan E., Baron M., Melendez S., Dave A. (2023). GABA Regulates Electrical Activity and Tumor Initiation in Melanoma. Cancer Discov..

[140] Elias A.F., Lin B.C., Piggott B.J. (2023). Ion Channels in Gliomas—From Molecular Basis to Treatment. Int. J. Mol. Sci..

[141] Gil D., Laidler P., Zarzycka M., Dulińska-Litewka J. (2021). Inhibition Effect of Chloroquine and Integrin-Linked Kinase Knockdown on Translation in Melanoma Cells. Int. J. Mol. Sci..

[142] Aloia A., Müllhaupt D., Chabbert C.D., Eberhart T., Flückiger-Mangual S., Vukolic A., Eichhoff O., Irmisch A., Alexander L.T., Scibona E. (2019). A Fatty Acid Oxidation-Dependent Metabolic Shift Regulates the Adaptation of BRAF-Mutated Melanoma to MAPK Inhibitors. Clin. Cancer Res. Off. J. Am. Assoc. Cancer Res..

[143] Chrzan N., Hartman M.L. (2025). Copper in Melanoma: At the Crossroad of Protumorigenic and Anticancer Roles. Redox Biol..

[144] Lionaki E., Ploumi C., Tavernarakis N. (2022). One-Carbon Metabolism: Pulling the Strings behind Aging and Neurodegeneration. Cells.

[145] Tincu (Iurciuc) C.-E., Andrițoiu C.V., Popa M., Ochiuz L. (2023). Recent Advancements and Strategies for Overcoming the Blood–Brain Barrier Using Albumin-Based Drug Delivery Systems to Treat Brain Cancer, with a Focus on Glioblastoma. Polymers.

[146] Yang S., Ta Y.-N.N., Chen Y. (2025). Nanotechnology-Enhanced Immunotherapies for Pancreatic Ductal Adenocarcinoma: Challenges and Opportunities. Drug Deliv. Transl. Res..

[147] Pallarés-Moratalla C., Bergers G. (2024). The Ins and Outs of Microglial Cells in Brain Health and Disease. Front. Immunol..

[148] Bogéa G.M.R., Silva-Carvalho A.É., Filiú-Braga L.D.d.C., Neves F.d.A.R., Saldanha-Araujo F. (2022). The Inflammatory Status of Soluble Microenvironment Influences the Capacity of Melanoma Cells to Control T-Cell Responses. Front. Oncol..

[149] Geltz A., Geltz J., Kasprzak A. (2025). Regulation and Function of Tumor-Associated Macrophages (TAMs) in Colorectal Cancer (CRC): The Role of the SRIF System in Macrophage Regulation. Int. J. Mol. Sci..

[150] Ma Z., Zhou F., Jin H., Wu X. (2024). Crosstalk between CXCL12/CXCR4/ACKR3 and the STAT3 Pathway. Cells.

[151] Covache-Busuioc R.-A., Toader C., Rădoi M.P., Șerban M. (2025). Precision Recovery After Spinal Cord Injury: Integrating CRISPR Technologies, AI-Driven Therapeutics, Single-Cell Omics, and System Neuroregeneration. Int. J. Mol. Sci..

[152] Kciuk M., Yahya E.B., Mohamed Ibrahim Mohamed M., Rashid S., Iqbal M.O., Kontek R., Abdulsamad M.A., Allaq A.A. (2023). Recent Advances in Molecular Mechanisms of Cancer Immunotherapy. Cancers.

[153] Arese M., Bussolino F., Pergolizzi M., Bizzozero L. (2022). An Overview of the Molecular Cues and Their Intracellular Signaling Shared by Cancer and the Nervous System: From Neurotransmitters to Synaptic Proteins, Anatomy of an All-Inclusive Cooperation. Int. J. Mol. Sci..

[154] Liu Y.-T., Wang Y.-L., Wang S., Li J.-J., He W., Fan X.-J., Wan X.-B. (2025). Turning Cold Tumors into Hot Tumors to Ignite Immunotherapy. Mol. Cancer.

[155] Habibi M.A., Mirjani M.S., Ahmadvand M.H., Delbari P., Eftekhar M.S., Ghazizadeh Y., Ghezel M.A., Rad R.H., Vakili K.G., Lotfi S. (2024). Anti-PD-1/PD-L1 Inhibitor Therapy for Melanoma Brain Metastases: A Systematic Review and Meta-Analysis. Neurosurg. Rev..

[156] Lanis M.R., Kim S., Schneck J.P. (2025). Hydrogels in the Immune Context: In Vivo Applications for Modulating Immune Responses in Cancer Therapy. Gels.

[157] Karkempetzaki A.I., Schatton T., Barthel S.R. (2025). Galectin-9—An Emerging Glyco-Immune Checkpoint Target for Cancer Therapy. Int. J. Mol. Sci..

[158] Koupourtidou C., Schwarz V., Aliee H., Frerich S., Fischer-Sternjak J., Bocchi R., Simon-Ebert T., Bai X., Sirko S., Kirchhoff F. (2024). Shared Inflammatory Glial Cell Signature after Stab Wound Injury, Revealed by Spatial, Temporal, and Cell-Type-Specific Profiling of the Murine Cerebral Cortex. Nat. Commun..

[159] Ye Q., Li Z., Li Y., Li Y., Zhang Y., Gui R., Cui Y., Zhang Q., Qian L., Xiong Y. (2022). Exosome-Derived microRNA: Implications in Melanoma Progression, Diagnosis and Treatment. Cancers.

[160] Doron H., Amer M., Ershaid N., Blazquez R., Shani O., Lahav T.G., Cohen N., Adler O., Hakim Z., Pozzi S. (2019). Inflammatory Activation of Astrocytes Facilitates Melanoma Brain Tropism via the CXCL10-CXCR3 Signaling Axis. Cell Rep..

[161] Sikorski H., Żmijewski M.A., Piotrowska A. (2025). Tumor Microenvironment in Melanoma—Characteristic and Clinical Implications. Int. J. Mol. Sci..

[162] Guan F., Wang R., Yi Z., Luo P., Liu W., Xie Y., Liu Z., Xia Z., Zhang H., Cheng Q. (2025). Tissue Macrophages: Origin, Heterogenity, Biological Functions, Diseases and Therapeutic Targets. Signal Transduct. Target. Ther..

[163] Toader C., Serban M., Covache-Busuioc R.-A., Radoi M.P., Aljboor G.S.R., Glavan L.-A., Corlatescu A.D., Ilie M.-M., Gorgan R.M. (2024). Navigating the Rare and Dangerous: Successful Clipping of a Superior Cerebellar Artery Aneurysm Against the Odds of Uncontrolled Hypertension. J. Clin. Med..

[164] Campbell K.M., Amouzgar M., Pfeiffer S.M., Howes T.R., Medina E., Travers M., Steiner G., Weber J.S., Wolchok J.D., Larkin J. (2023). Prior Anti-CTLA-4 Therapy Impacts Molecular Characteristics Associated with Anti-PD-1 Response in Advanced Melanoma. Cancer Cell.

[165] Balbi M., Bonanno G., Bonifacino T., Milanese M. (2023). The Physio-Pathological Role of Group I Metabotropic Glutamate Receptors Expressed by Microglia in Health and Disease with a Focus on Amyotrophic Lateral Sclerosis. Int. J. Mol. Sci..

[166] Hodo T.W., de Aquino M.T.P., Shimamoto A., Shanker A. (2020). Critical Neurotransmitters in the Neuroimmune Network. Front. Immunol..

[167] Zhang Y., Wang N., Zhang L., Zhuang Y., Xin Q., Gu X., Jiang C., Wu J. (2025). Serotonin (5-Hydroxytryptamine): Metabolism, Signaling, Biological Functions, Diseases, and Emerging Therapeutic Opportunities. MedComm.

[168] Yang J., Wu Y., Lv X., Liu S., Yuan Z., Chen Y., Ding X., Li Z., Wang X. (2025). Neurotransmitters: An Emerging Target for Therapeutic Resistance to Tumor Immune Checkpoint Inhibitors. Mol. Cancer.

[169] Rodriguez-Baena F.J., Marquez-Galera A., Ballesteros-Martinez P., Castillo A., Diaz E., Moreno-Bueno G., Lopez-Atalaya J.P., Sanchez-Laorden B. (2025). Microglial Reprogramming Enhances Antitumor Immunity and Immunotherapy Response in Melanoma Brain Metastases. Cancer Cell.

[170] Poljsak B., Kovac V., Dahmane R., Levec T., Starc A. (2019). Cancer Etiology: A Metabolic Disease Originating from Life’s Major Evolutionary Transition?. Oxid. Med. Cell. Longev..

[171] Internò V., Sergi M.C., Metta M.E., Guida M., Trerotoli P., Strippoli S., Circelli S., Porta C., Tucci M. (2023). Melanoma Brain Metastases: A Retrospective Analysis of Prognostic Factors and Efficacy of Multimodal Therapies. Cancers.

[172] Prakash R., Izraely S., Thareja N.S., Lee R.H., Rappaport M., Kawaguchi R., Sagi-Assif O., Ben-Menachem S., Meshel T., Machnicki M. (2019). Regeneration Enhances Metastasis: A Novel Role for Neurovascular Signaling in Promoting Melanoma Brain Metastasis. Front. Neurosci..

[173] Toader C., Brehar F.M., Radoi M.P., Covache-Busuioc R.A., Serban M., Ciurea A.V., Dobrin N. (2024). Challenging Management of a Rare Complex Cerebral Arteriovenous Malformation in the Corpus Callosum and Post-Central Gyrus: A Case Study of a 41-Year-Old Female. J. Clin. Med..

[174] Jacome M.A., Wu Q., Chen J., Mohamed Z.S., Mokhtari S., Piña Y., Etame A.B. (2025). Molecular Underpinnings of Brain Metastases. Int. J. Mol. Sci..

[175] Isaak A.J., Clements G.R., Buenaventura R.G.M., Merlino G., Yu Y. (2024). Development of Personalized Strategies for Precisely Battling Malignant Melanoma. Int. J. Mol. Sci..

[176] Tittarelli A., Pereda C., Gleisner M.A., López M.N., Flores I., Tempio F., Lladser A., Achour A., González F.E., Durán-Aniotz C. (2024). Long-Term Survival and Immune Response Dynamics in Melanoma Patients Undergoing TAPCells-Based Vaccination Therapy. Vaccines.

[177] Putnam A.J., Schulz V.V., Freiter E.M., Bill H.M., Miranti C.K. (2009). Src, PKCα, and PKCδ Are Required for Avβ3 Integrin-Mediated Metastatic Melanoma Invasion. Cell Commun. Signal..

[178] Dang N.-N., Li X.-B., Zhang M., Han C., Fan X.-Y., Huang S.-H. (2021). NLGN3 Upregulates Expression of ADAM10 to Promote the Cleavage of NLGN3 via Activating the LYN Pathway in Human Gliomas. Front. Cell Dev. Biol..

[179] Matsuzaka Y., Yashiro R. (2024). Classification and Molecular Functions of Heparan Sulfate Proteoglycans and Their Molecular Mechanisms with the Receptor. Biologics.

[180] Li K., Duan M., Lu Q., Liu J., He M., Zhang Y. (2025). Advances in Neuroscientific Mechanisms and Therapies for Glioblastoma. iScience.

[181] Sun H., Wang T., Jiang X., Li M., He X., Ma Y., Li X., Jin W., Jiao Z. (2025). Integrating Neuroscience and Oncology: Neuroimmune Crosstalk in the Initiation and Progression of Digestive System Tumors. Mol. Cancer.

[182] Koda S., Hu J., Ju X., Sun G., Shao S., Tang R.-X., Zheng K.-Y., Yan J. (2023). The Role of Glutamate Receptors in the Regulation of the Tumor Microenvironment. Front. Immunol..

[183] Boxer E.E., Aoto J. (2022). Neurexins and Their Ligands at Inhibitory Synapses. Front. Synaptic Neurosci..

[184] Neumann E.K. (2025). Spatial Multiomics Toward Understanding Neurological Systems. J. Mass Spectrom..

[185] Tomaszewski W.H., Waibl-Polania J., Chakraborty M., Perera J., Ratiu J., Miggelbrink A., McDonnell D.P., Khasraw M., Ashley D.M., Fecci P.E. (2022). Neuronal CaMKK2 Promotes Immunosuppression and Checkpoint Blockade Resistance in Glioblastoma. Nat. Commun..

[186] Koltai T., Fliegel L. (2024). Exploring Monocarboxylate Transporter Inhibition for Cancer Treatment. Explor. Target. Anti-Tumor Ther..

[187] Koren T., Rolls A. (2022). Immunoception: Defining Brain-Regulated Immunity. Neuron.

[188] Wang J.S., Yassin S., Lin A.Y. (2025). Utilization of Exosome-Based Therapies to Augment Anti-PD-1/PD-L1 Therapies. J. Immunother. Cancer.

[189] León-Rodríguez A., Grondona J.M., Marín-Wong S., López-Aranda M.F., López-Ávalos M.D. (2025). Long-Term Reprogramming of Primed Microglia after Moderate Inhibition of CSF1R Signaling. Glia.

[190] Lin J.-F., Wang T.-T., Huang R.-Z., Tan Y.-T., Chen D.-L., Ju H.-Q. (2025). PANoptosis in Cancer: Bridging Molecular Mechanisms to Therapeutic Innovations. Cell. Mol. Immunol..

[191] Pujalte-Martin M., Belaïd A., Bost S., Kahi M., Peraldi P., Rouleau M., Mazure N.M., Bost F. (2024). Targeting Cancer and Immune Cell Metabolism with the Complex I Inhibitors Metformin and IACS-010759. Mol. Oncol..

[192] Nardin C., Peres C., Putti S., Orsini T., Colussi C., Mazzarda F., Raspa M., Scavizzi F., Salvatore A.M., Chiani F. (2021). Connexin Hemichannel Activation by S-Nitrosoglutathione Synergizes Strongly with Photodynamic Therapy Potentiating Anti-Tumor Bystander Killing. Cancers.

[193] Șerban M., Toader C., Covache-Busuioc R.-A. (2025). Brain Tumors, AI and Psychiatry: Predicting Tumor-Associated Psychiatric Syndromes with Machine Learning and Biomarkers. Int. J. Mol. Sci..

[194] Toader C., Serban M., Eva L., Costea D., Covache-Busuioc R.-A., Radoi M.P., Ciurea A.V., Dumitru A.V. (2025). Large Pontine Cavernoma with Hemorrhage: Case Report on Surgical Approach and Recovery. J. Clin. Med..

[195] Guerra A., Bologna M. (2022). Low-Intensity Transcranial Ultrasound Stimulation: Mechanisms of Action and Rationale for Future Applications in Movement Disorders. Brain Sci..

[196] Parker A.L., Benguigui M., Fornetti J., Goddard E., Lucotti S., Insua-Rodríguez J., Wiegmans A.P. (2022). Current Challenges in Metastasis Research and Future Innovation for Clinical Translation. Clin. Exp. Metastasis.

[197] Bravo F., Glogowski J., Stamatakis E.A., Herfert K. (2024). Dissonant Music Engages Early Visual Processing. Proc. Natl. Acad. Sci. USA.

